# Improved TMC1 gene therapy restores hearing and balance in mice with genetic inner ear disorders

**DOI:** 10.1038/s41467-018-08264-w

**Published:** 2019-01-22

**Authors:** Carl A. Nist-Lund, Bifeng Pan, Amy Patterson, Yukako Asai, Tianwen Chen, Wu Zhou, Hong Zhu, Sandra Romero, Jennifer Resnik, Daniel B. Polley, Gwenaelle S. Géléoc, Jeffrey R. Holt

**Affiliations:** 1 Department of Otolaryngology and F.M. Kirby Neurobiology Center, Boston Children’s Hospital, 300 Longwood Avenue, Boston, MA 02115 USA; 2 Department of Otolaryngology, Harvard Medical School, Boston, MA 02139 USA; 3 Department of Otolaryngology and Communicative Sciences, University of Mississippi Medical Center, Jackson, MS 39216 USA; 4 Eaton-Peabody Laboratories, Massachusetts Eye and Ear Infirmary, Boston, MA 02139 USA; 5 Department of Neurology, Boston Children’s Hospital, Harvard Medical School, Boston, MA 02115 USA

## Abstract

Fifty percent of inner ear disorders are caused by genetic mutations. To develop treatments for genetic inner ear disorders, we designed gene replacement therapies using synthetic adeno-associated viral vectors to deliver the coding sequence for Transmembrane Channel-Like (*Tmc*) 1 or 2 into sensory hair cells of mice with hearing and balance deficits due to mutations in *Tmc1* and closely related *Tmc2*. Here we report restoration of function in inner and outer hair cells, enhanced hair cell survival, restoration of cochlear and vestibular function, restoration of neural responses in auditory cortex and recovery of behavioral responses to auditory and vestibular stimulation. Secondarily, we find that inner ear *Tmc* gene therapy restores breeding efficiency, litter survival and normal growth rates in mouse models of genetic inner ear dysfunction. Although challenges remain, the data suggest that *Tmc* gene therapy may be well suited for further development and perhaps translation to clinical application.

## Abstract (web summary)

Mutations in the mechanotransduction channel component TMC1/2 cause deafness. Here, the authors use a synthetic AAV to replace TMC1 and 2 in the inner ear and show restoration of cochlear and vestibular function, of neuronal reponses in the auditory cortex and of hearing and balance in mice.

## Introduction

Hearing loss is the most common neurological disorder and affects an estimated 466 million people worldwide, 35 million of which are children (World Health Organization). In addition to direct loss of auditory function, a number of secondary consequences can lead to diminished quality of life^1^, including impaired cognitive development in children, social isolation, depression, and dementia^2,3^. Despite the devastating consequences of hearing loss, there are no biological treatments for auditory dysfunction. The current standards of care provide only partial restoration for a limited patient population and include hearing aids, which boost sound levels and require some residual function, and cochlear implants, which bypass the sensory organ and directly stimulate the eighth cranial nerve.

In addition to the auditory organ, the inner ear includes five vestibular organs which mediate sensitivity to linear head moment and gravity, as well as rotational head movements around three orthogonal axes. Together, the vestibular end organs help maintain stable posture and gaze and contribute to the sense of balance. Loss of vestibular function can lead to vertigo (i.e., the illusion of motion), imbalance, blurred vision and orthostatic intolerance. Vestibular dysfunction is a risk factor for falling, which is one of the most common reasons elderly patients seek medical attention^4^. Unfortunately, there are no biological treatments for genetic vestibular dysfunction.

In a continuing effort to develop biological treatments for inner ear dysfunction, here we focus on development of improved gene therapy strategies to restore function in mouse models of genetic inner ear disorders. In particular, we focus on transmembrane channel-like 1 (*TMC1*) which encodes a protein required for the proper function and survival of inner ear hair cells. Recent evidence demonstrates that TMC1, and probably TMC2, are pore-forming components of mechanosensory transduction channels in auditory and vestibular hair cells^5,6^. Mutations in *Tmc1/TMC1* cause both dominant and recessive forms of deafness in mice and humans^7,8^. Estimates suggest three to eight percent of genetic hearing loss may be due to *TMC1* mutations^9,10^. Some recessive *TMC1* mutations cause moderate to severe hearing loss with post-lingual onset^10,11^. These patients may retain viable hair cells amenable to gene therapy intervention.

Prior gene therapy strategies that targeted *TMC1* focused on gene replacement using conventional adeno-associated viral (AAV) vector serotypes to restore function in mouse models of recessive *TMC1* deafness, DFNB7/11^12^, gene silencing using either AAV delivery of microRNAs^13^ or ribonucleotide protein complexes to deliver CRISPR/Cas9^14^ to target dominant *TMC1* mutations. While these therapeutic strategies revealed no adverse effects, they yielded only partial recovery of auditory function. The recovery was limited due to inefficient delivery of the therapeutic DNA constructs to the targeted cell types, inner and outer hair cells of the cochlea. Although these approaches demonstrated successful targeting of IHCs, efficient targeting of OHCs has been problematic for these and other studies^15,16^. Outer hair cells (OHCs) amplify and tune auditory information, which is transduced and transmitted by inner hair cells (IHCs). Both cell types are required for the fully functional cochlea, which enables exquisite sensitivity to faint sounds and fine pitch perception.

To address the limited recovery of auditory function with conventional AAV vectors, we previously screened a number of viral capsids and identified a synthetic AAV (sAAV), known as Anc80L065, which transduced IHCs and OHCs with high efficiency^17^. In prior work, the Anc80L065 capsid was shown to efficiently transduce a variety of cell types, including, retina, liver, and muscle^18^. In the ear, we used the Anc80L065 capsid in a gene therapy study focused on Usher Syndrome, type IC, and found an unprecedented level of auditory recovery in deaf mice exposed to sAAVs encoding the wild-type Usher 1 C gene^19^.

For the current study we used a mouse model of DFNB7/11 recessive deafness to investigate whether sAAVs encoding *Tmc1* can enhance auditory recovery, beyond that previously reported for *Tmc1* packaged in conventional AAV1 capsids^12^. In addition, we ask whether restoration of function in the auditory periphery can drive neuronal function at the level of auditory cortex, which would be required for perception of complex sounds and speech. We also investigate the ability of exogenous *Tmc1* and *Tmc2* to restore vestibular and balance function in mouse models of genetic vestibular dysfunction. Since both normal hearing and balance contribute to quality of life in humans, we investigate whether sAAVs encoding TMC proteins improve secondary measures of recovery in mouse models of inner ear dysfunction. Although challenges remain^15,16^, we conclude that efficient delivery of wild-type *Tmc1* restores hearing, balance and several secondary outcomes, suggesting inner ear gene therapy for *TMC1* mutations may be well-suited for further development and perhaps translation to the clinic.

## Results

### Tmc1 gene therapy restores sensory transduction

In prior work, we assessed *Tmc1* gene therapy using an AAV2/1 serotype with a chicken beta actin (*Cba*) promoter driving expression of wild-type *Tmc1* in a mouse model of DFNB7/11^12^. We reported recovery of sensory transduction in inner hair cells (IHCs), but little or no recovery in outer hair cells (OHCs), most likely due to lack of expression or targeting in OHCs of AAV2/1-injected cochleas^12^. As a result, recovery of auditory function was modest. More recently, we demonstrated that synthetic AAV2/Anc80L65, together with a cytomegalovirus (*Cmv*) promoter, efficiently expressed eGFP in both IHCs and OHCs when injected via the round window membrane (RWM) at early postnatal stages^17^. For the present study, we combined the two strategies and generated gene therapy vectors that encoded a *Cmv* promoter, an identical therapeutic gene, *Tmc1ex1*, followed by a *WPRE* sequence, packaged into synthetic AAV2/Anc80L65 capsids: AAV2/Anc80L65-*Cmv*-*Tmc1ex1-WPRE* (for brevity: sAAV-*Tmc1*). We injected 1 μL of sAAV-*Tmc1* (1.4 × 10^14^ gc/mL) between postnatal day (P) 0 and P2 via RWM of *Tmc1*^∆/∆^;*Tmc2*^∆/∆^ mice.

**Fig. 1:**
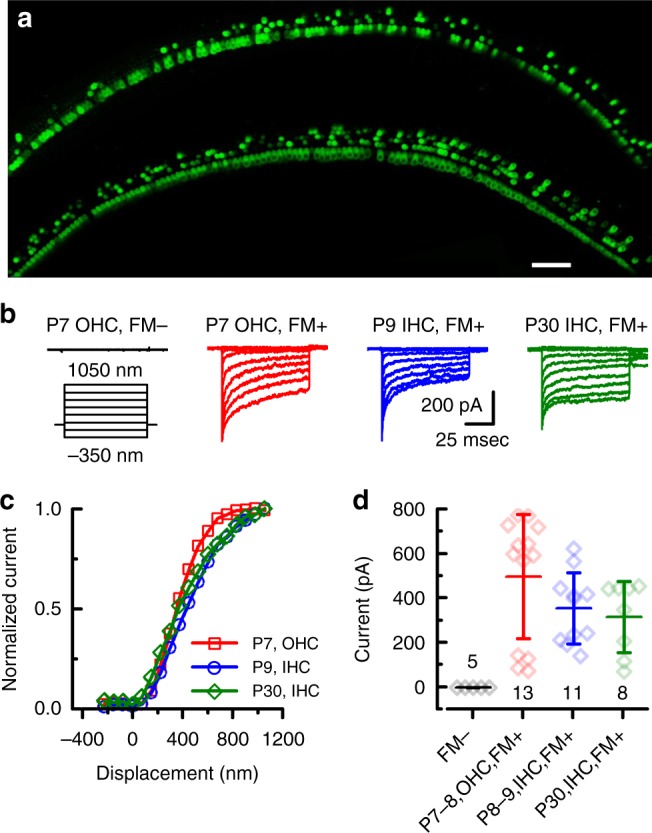
sAAV-*Tmc1* restores sensory transduction in *Tmc1*^*∆/∆*^*;Tmc2*^*∆/∆*^ IHCs and OHCs. **a** Confocal images of two apical sections excised from P7 *Tmc1*^*∆/∆*^*;Tmc2*^*∆/∆*^ mouse cochleas after injection of sAAV-*Tmc1* through the RWM at P1. The tissue was cultured for 7 days and perfused with 5 µM FM1-43 for 10 s followed by 3 full bath exchanges to washout excess dye. The tissue was then mounted and imaged for FM1-43 uptake (green) in IHCs and OHCs. Scale bar = 25 µm. **b** Representative families of sensory transduction currents evoked by mechanical displacement of hair bundles (protocol shown below) recorded from P7 apical OHCs of *Tmc1*^*∆/∆*^*;Tmc2*^*∆/∆*^ mice injected with sAAV-*Tmc1* that were FM1-43-negative (left) or FM1-43-positive (second from left). FM1-43-positive currents were also recorded from P9 IHCs (second from right) and P30 IHCs (right) of *Tmc1*^*∆/∆*^*;Tmc2*^*∆/∆*^ mice injected with sAAV-*Tmc1*. **c** Stimulus-response curves from the currents shown in **b**. **d** Mean ± S.D. peak sensory transduction current amplitudes from apical FM1-43-negative and FM1-43-positive IHCs and OHCs from *Tmc1*^*∆/∆*^*;Tmc2*^*∆/∆*^ mice injected with sAAV-*Tmc1* at time points as indicated. *n* = number of hair cells

To determine the efficacy of viral targeting, expression of exogenous *Tmc1*, and restoration of sensory transduction in auditory hair cells, we visualized uptake of FM1-43, a styryl dye which permeates hair cell sensory transduction channels^20,21^. Since both *Tmc1* and *Tmc2* are expressed at early postnatal stages (<P10)^22^, we injected inner ears of *Tmc1*^∆/∆^;*Tmc2*^∆/∆^ mice which lack expression of endogenous TMC1 and TMC2 proteins, and thus do not take up FM1-43^12,22^. Likewise, age-matched, sham injected *Tmc1*^∆/∆^;*Tmc2*^∆/∆^ mice also lacked FM1-43 uptake. However, in *Tmc1*^∆/∆^;*Tmc2*^∆/∆^ mice seven days post-injection with sAAV-*Tmc1*, we found robust FM1-43 uptake in most IHCs (91 ± 8%, *n* = 4 cochleas) and half of the OHCs (50 ± 17%), suggesting expression of exogenous TMC1 and the presence of functional sensory transduction (Fig. 1a).

To assess sensory transduction currents we recorded from IHCs and OHCs of *Tmc1*^∆/∆^;*Tmc2*^∆/∆^ mice injected on P1 with sAAV-*Tmc1*. Cochleas were harvested on P5-P7 and maintained in organotypic culture. On the equivalent of P7, *Tmc1*^∆/∆^;*Tmc2*^∆/∆^ hair cells which did not take up FM1-43, lacked sensory transduction (Fig. 1b, left). FM1-43-positive OHCs within the same culture displayed robust sensory transduction (Fig. 1b, center left). Additionally, IHCs displayed sensory transduction at the equivalent of P8-9 (Fig. 1b, center right), and these responses were maintained to the equivalent of P30 (Fig. 1b, right), the latest time point tested. Hair cells from all three experimental groups displayed sensitivity, as indicated from stimulus-response relationships (Fig. 1c), and sensory transduction current amplitudes (Fig. 1d) that were not significantly different (*p* = 0.45, one-way ANOVA at P30 + IHCs) from wild-type hair cells and those of wild-type cells transduced with sAAV-*Cmv*-GFP^17^. The hair cell physiology data together with the FM1-43 data indicate that in vivo delivery of sAAV-*Tmc1* promotes recovery of sensory transduction in large populations of IHCs and OHCs. In addition, the IHC currents recorded at P30 demonstrate gene therapy rescue of sensory function in mature hair cells, after the onset of hearing.

### Tmc1 gene therapy restores cochlear function

**Fig. 2:**
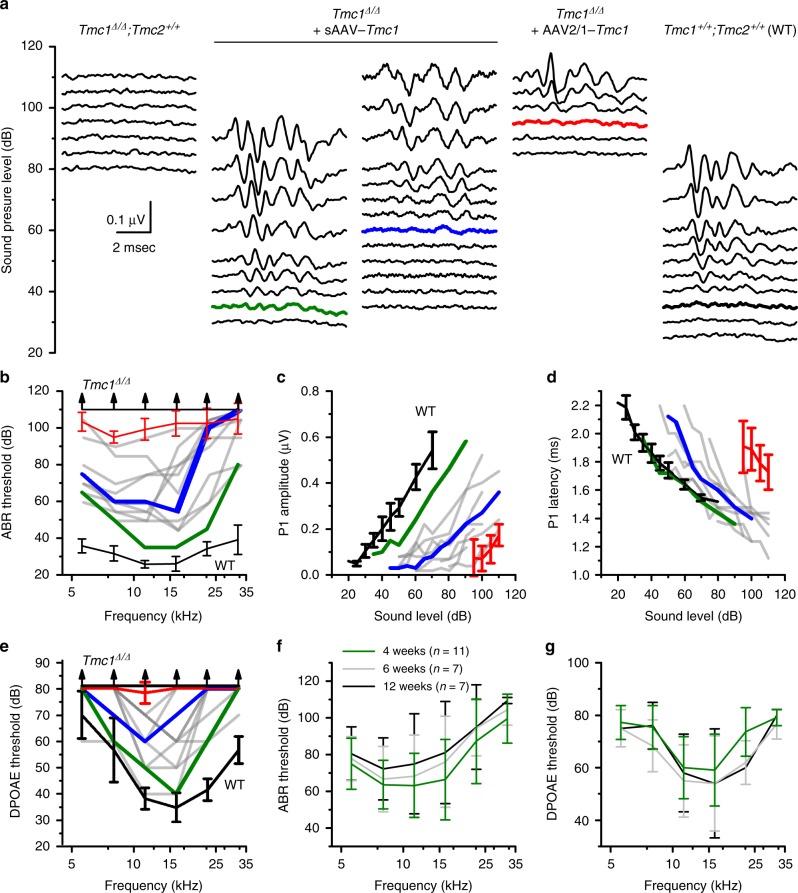
sAAV-*Tmc1* restores ABRs and DPOAEs in *Tmc1*^*∆/∆*^ mice. **a** Families of ABR waveforms recorded at P28-P30 from uninjected *Tmc1*^*∆/∆*^ mouse (left) and from *Tmc1*^*∆/∆*^ mice injected with sAAV-*Tmc1* or AAV2/1-*Tmc1* and one WT control, as indicated above. ABRs were recorded using 11.3-kHz tone bursts at sound pressure levels increasing by 5-dB until peak amplitudes reached 0.55 μV. Thresholds determined by the presence of Peak 1 and is indicated by colored traces. Scale bar applies to all families. **b** ABR thresholds plotted as a function of stimulus frequency for eleven *Tmc1*^*∆/∆*^ mice injected with sAAV-*Tmc1* tested at P28-P30 (gray traces). Best (green), and median (blue) recovery are indicated. Black lines show mean ± S.D. (*n* *=* 6 mice) for WT and *Tmc1*^*∆/∆*^ uninjected mice as indicated. Red line shows mean ± S.D. (*n* *=* 6 mice) for *Tmc1*^*∆/∆*^ injected with AAV2/1-*Tmc1*. **c** Peak 1 amplitudes measured from 11.3-kHz ABR waveforms, as shown in **a**, for eleven *Tmc1*^∆/∆^ mice injected with sAAV-*Tmc1*. Colors correspond to conditions indicated in **b**. **d** Peak 1 latencies measured from 11.3-kHz ABR waveforms, as shown in **a** for eleven *Tmc1*^*∆/∆*^ mice injected with sAAV-*Tmc1*. Colors correspond to conditions indicated in **b**. **e** DPOAE thresholds plotted as a function of stimulus frequency for eleven *Tmc1*^*∆/∆*^ mice injected with sAAV-*Tmc1* tested at P28-P30. Colors correspond to conditions indicated in **b**. **f** Mean ± S.D. (*n* = number of mice) ABR thresholds plotted as a function of stimulus frequency for *Tmc1*^*∆/∆*^ mice injected with sAAV-*Tmc1* at P1 tested at 4, 6, and 12 weeks. **g** Mean ± S.D. DPOAE thresholds plotted as a function of stimulus frequency for the same *Tmc1*^*∆/∆*^ mice shown in panel G injected with sAAV-*Tmc1*

To investigate the extent of recovery from sAAV-*Tmc1* at the level of the whole cochlea, we measured auditory brainstem responses (ABRs) and distortion product otoacoustic emissions (DPOAEs) beginning at P28. Since TMC2 is not expressed in cochlear hair cells at mature stages^22,23^, we used *Tmc1*^*∆/∆*^ mice, which in the absence of gene therapy are profoundly deaf. *Tmc1*^*∆/∆*^ *m*ice were injected at P1-P2, and cochlear function was assessed at P28 to P30. Figure 2a shows representative families of ABR waveforms recorded in response to 11.3-kHz tone bursts of varying sound intensity. The families were selected to illustrate responses of an uninjected *Tmc1*^*∆/∆*^ mouse (left), two *Tmc1*^∆/∆^ mice injected with sAAV-*Tmc1* with ABRs that displayed the lowest and median thresholds, a *Tmc1*^∆/∆^ mouse injected with 1 µL of AAV2/1-*Cmv*-*Tmc1* (8.1 × 10^14^ gc/mL), and an aged-matched *Tmc1*^*+/+*^ wild-type control (right). Of 11 mice injected with sAAV-*Tmc1*, all showed improvement in auditory function relative to uninjected *Tmc1*^*∆/∆*^ mice and relative to the mean response of six *Tmc1*^*∆/∆*^ mice injected with AAV2/1-*Tmc1* (Fig. 2b). Variability in the extent of the recovery may have been due to injection variability, local variability of viral titer within the cochlea or variability of transduction efficiency. The variability is consistent with prior reports of variable gene therapy recovery^15,16^. The source and methods for reducing variability in mouse gene therapy studies will require additional study.

ABR thresholds for the 11 *Tmc1*^*∆/∆*^ mice injected with sAAV-*Tmc1* ranged from 35 to 90 dB and corresponded to best frequencies of 8–16 kHz (Fig. 2b). ABR thresholds of sAAV-*Tmc1-*injected mice were significantly lower than the six *Tmc1*^*∆/∆*^ mice injected with AAV2/1-*Tmc1* at all frequencies tested between 5 and 16 kHz (*p* ≤ 0.00024, paired *t*-test). ABR peak I amplitudes were generally monotonic increasing functions of sound level (Fig. 2c), while peak I latencies were monotonic decreasing functions of sound intensity (Fig. 2d), both consistent with curves of normal hearing mice. We also injected sAAV-*Tmc1* into the inner ears of four WT C57BL/6 mice and found no change in auditory function relative to uninjected WT controls, which suggested the injection technique, the viral capsid and overexpression of *Tmc1* had no detrimental effect on inner ear function (Supplementary Fig. 1).

To assess OHC function at the level of the whole cochlea, we measured DPOAEs, a proxy for cochlear amplification and a requirement for sensitivity to soft sounds and fine frequency selectivity. In prior work, we found no recovery of DPOAEs in *Tmc1*^∆/∆^ mice injected with AAV2/1*-Cba-Tmc1*^12^. For the current study, we found that AAV2/1*-Cmv-Tmc1* injected mice also lacked recovery of DPOAEs, likely the result of limited viral transduction in OHCs with the AAV2/1 serotype. However, following sAAV-*Tmc1* injection, all 11 *Tmc1*^*∆/∆*^ mice had improved DPOAE responses (Fig. 2e), relative to uninjected *Tmc1*^∆/∆^ mice, consistent with more efficient OHC transduction and the enhanced ABR thresholds. Both the recovery of ABR and DPOAE thresholds were durable over time with no significant (*p* > 0.05) differences at four, six, and twelve weeks of age (Fig. 2f, g).

Since the cochlear aqueduct, which provides a fluid connection between the inner ear and cerebral spinal fluid, is patent in neonatal mice, we examined cochlear function in contralateral ears of *Tmc1*^∆/∆^ mice injected with sAAV-*Tmc1* on the ipsilateral side. Contralateral ears displayed recovery of ABR thresholds, albeit to a lesser extent than injected ears (Supplementary Fig. 2). The performance of the contralateral ear was not correlated with the level of performance in the injected ear, likely due to lower viral distribution to the contralateral ear. However, in contralateral ears with recovery, ABR and DPOAE thresholds were durable from 6 to 12 weeks, similar to the durable recovery observed in injected ears.

To investigate ability of sAAV-*Tmc1* to restore function at later time points, we injected *Tmc1*^*∆/∆*^ mice at P1, P4, P7, and P14. ABR and DPOAE thresholds were recorded at P28. We found elevated ABR thresholds and little or no recovery of DPOAEs following RWM injection at later stages (Supplementary Fig. 3). To investigate whether the lack of recovery was due to decreased expression of exogenous transgene we used the same RWM injection technique, same viral capsid and promoter, but swapped out the *Tmc1* coding sequence for the green fluorescent protein (*eGFP*) coding sequence. We found the percentage of GFP-positive hair cells decreased as a function of injection age (from 93% at P1 to 3% at P14; Supplementary Fig. 4), suggesting that either viral capsid or promoter efficiency was reduced at later stages. Development of viral capsids, promoters, or injection techniques that drive exogenous gene expression in IHCs and OHCs when injected at mature stages will require further investigation. Once this limitation is overcome, whether exogenous *Tmc1* can restore hair cell and auditory function at later stages will need to be addressed (however, see vestibular section below).

### Tmc1 gene therapy promotes hair cell survival

**Fig. 3:**
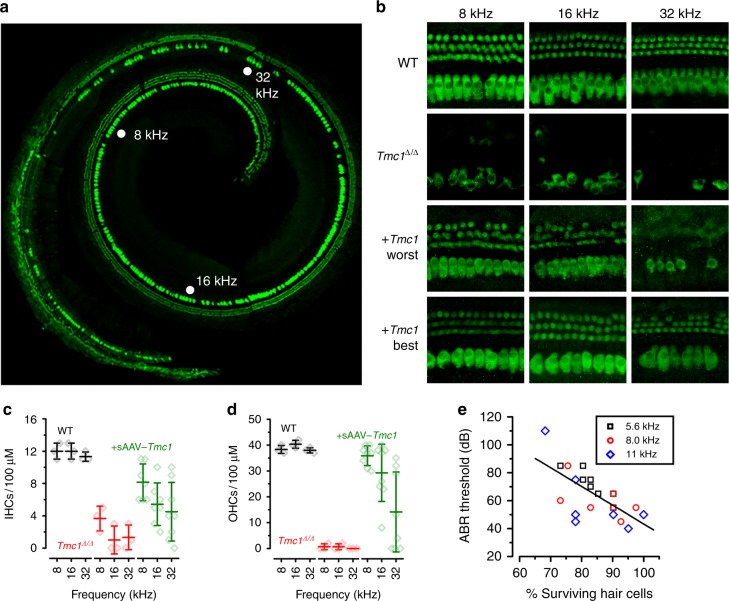
sAAV-*Tmc1* promotes survival of IHCs and OHCs in *Tmc1*^*∆/∆*^ mice. **a** Composite of five images from a whole cochlea harvested at 12 weeks old from a *Tmc1*^*∆/∆*^ mouse injected with sAAV-*Tmc1* on P1 reconstructed from microdissected tissue. The tissue was stained for Myo7a (green). **b** Tonotopically mapped cochlear sections (image width = 100 µm) at 8, 16, and 32 kHz regions for WT (top), *Tmc1*^*∆/∆*^ (second), and *Tmc1*^*∆/∆*^-injected with sAAV-*Tmc1* on P1 with the worst (third) and best (bottom) ABR performances as indicated in Fig. 2. **c**, **d** The number of surviving IHCs (**c**) and OHCs (**d**) in sections indicated in **b** from 3 WT  mice, 3 uninjected *Tmc1*^*∆/∆*^, and eight *Tmc1*^*∆/∆*^ mice injected with sAAV-*Tmc1* on P1 showing mean ± S.D. Number of surviving IHCs in the 8 and 16 kHz was significantly greater in injected ears than in uninjected *Tmc1*^*∆/∆*^ ears at (*t*-test: *p* = 0.015, 0.03, 0.19, respectively). Number of surviving OHCs in the 8 and 16 kHz was significantly greater in injected ears than in uninjected *Tmc1*^*∆/∆*^ ears at (*t*-test: *p* = 1E−7, 0.002, 0.16, respectively). **e** The percentage of surviving hair cells at 12 weeks of age was correlated with ABR thresholds measured between 5.6 and 11 kHz from sAAV-*Tmc1-*injected *Tmc1*^*∆/∆*^ mice. The data were fit with a linear regression with *r* = −0.67 and *p* = 6.2 × 10^-4^ (*n* = 7 mice; 21 measurements)

Both inner and outer hair cells of *Tmc1*^∆/∆^ mice begin to die around four weeks of age, progressing from the base of the cochlea toward the apex^22^. To investigate the ability of sAAV-*Tmc1* to promote hair cell survival, *Tmc1*^∆/∆^ mice injected with sAAV-*Tmc1* at P1 were euthanized at twelve weeks of age, and their inner ear tissue was excised for histological examination. There was no overt evidence of inflammation, tissue damage, or decay in any of the injected ears. Cochleas were harvested and the entire organ of Corti was dissected, mounted and stained with an anti-Myo7A antibody to label hair cells. Cochlear fragments were imaged and tonotopically mapped across the entire cochlea. Representative composite images of full cochleas reconstructed from five cochlear fragments are shown in Fig. 3a & Supplementary Figure 5 with the 8, 16, and 32 kHz regions indicated. Hundred-micron sections, centered at these frequency regions, were imaged at higher magnification (Fig. 3b). There was significant hair cell loss in the *Tmc1*^*∆/∆*^ cochleas, with almost no surviving OHCs in any region examined, consistent with prior reports^22^. However, in all injected cochleas examined, we observed enhanced survival of both IHCs and OHCs. Surviving hair cells were counted and are plotted in Fig. 3c, d. The data reveal examples of enhanced survival in all three regions with better survival, on average, at the low frequency end of the spectrum, consistent with enhanced ABR and DPOAE recovery at lower frequencies (Fig. 2f, g). Furthermore, we found that the number of surviving hair cells correlated (*r* = 0.67) with lower ABR thresholds in the low frequency region of the cochlea, between 5.6 and 11 kHz (Fig. 3e).

### Tmc1 gene therapy restores responses in auditory cortex

Since several genes that cause hearing loss when mutated are known to be expressed in the cochlea and in central auditory pathways^24,25^, we asked whether recovery of peripheral auditory function in sAAV-*Tmc1-*injected *Tmc1*^*∆/∆*^ mice is sufficient to drive neural responses in auditory cortex.

**Fig. 4:**
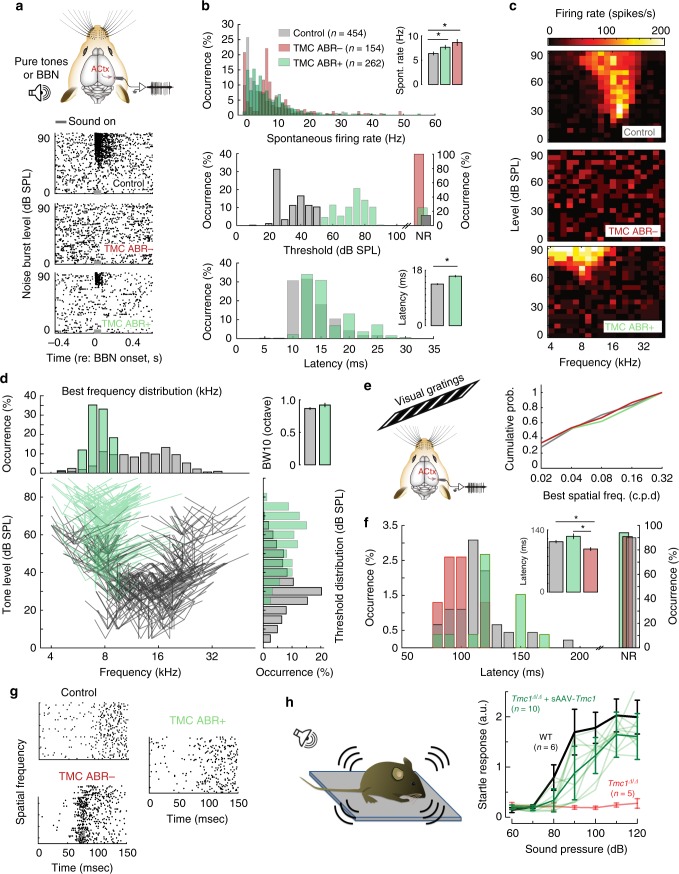
sAAV-*Tmc1* restores neuronal responses in auditory cortex of *Tmc1*^*∆/∆*^ mice. **a** (Top) Schematic of multi-unit (MU) activity recordings in the auditory cortex. (Below) Representative raster plots from each group. Data were recorded from six C57B/L6 wild-type control mice and eleven awake head-fixed *Tmc1*^*∆/∆*^ mice injected with sAAV-*Tmc1. Tmc1*^*∆/∆*^-injected mice were divided into two groups, those with significant improvement in ABR thresholds (ABR+: thresholds ≤70 dB at any frequency; *n* = 7 mice) and mice with little improvement in thresholds (ABR−: thresholds ≥75 dB at all frequencies; *n* = 4 mice). Sound stimulus duration indicated as gray bar. BBN: broad band noise, ACtx: auditory cortex. **b** Quantification of spontaneous activity for WT control mice and *Tmc1*^*∆/∆*^-injected mice (top). Quantification of both threshold (middle) and latency (bottom) resulting from broad band noise (BBN) stimuli presentation at different sound levels. **c** Representative frequency response areas after presentation of 361 frequency-level sound combinations. **d** Distribution of population tuning properties for responsive units Illustrating the best frequency (tip) and bandwidth measured 10 dB above threshold (side lines). Distribution of pure tone thresholds (bottom, right) and best frequencies (top, left) for responsive units. (Top, right) Bandwidth tuning responses for the stimulus presentation in (C). **e** (Top left) Schematic of recordings from auditory cortex during presentation of a visual stimulus consisting of gratings with changing spatial frequency. (Right) Preferred spatial frequency among ABR+, ABR−, and control mice. **f** Quantification of visually evoked onset latency in ABR− mice, ABR+ and control mice. **g** Representative raster plots from neurons recorded from the paradigm in **f**. **h** Startle response amplitudes normalized to mouse weight measured at P28-P30 and plotted as a function of sound intensity and as mean ± S.D. for 6 control C57BL/6 mice (black), ten individual *Tmc1*^*∆/∆*^ mice injected with sAAV-*Tmc1* (light green, mean ± S.D. in bold green), and five uninjected *Tmc1*^*∆/∆*^ mice (red)

To investigate central auditory function after sAAV-*Tmc1* gene therapy, we recorded multi-unit (MU) activity in the auditory cortex (ACtx) of awake, head-fixed *Tmc1*^*∆/∆*^ mice (Fig. 4a). Eleven *Tmc1*^*∆/∆*^ mice were divided into two groups, seven sAAV-*Tmc1*-injected mice with improvement in ABR thresholds (ABR+: thresholds ≤70 dB at any frequency) and four sAAV-*Tmc1*-injected mice with minimal improvement in thresholds (ABR-: thresholds ≥75 dB). The variability in ABR thresholds was likely due to the success of the injection technique. We found that ACtx neurons of both ABR+ and ABR− mice had higher spontaneous activity than C57BL/6 wild-type control mice (Figs. 4a top, b, Kruskal–Wallis test *χ*^2^(2) = 32.22; pairwise comparisons: control vs. ABR+, *p* = 2.1e−05; control vs. ABR−, *p* = 8.5e−06). To test whether cochlear gene therapy supported sound processing in regions of the central auditory pathway, we presented broad band noise (BBN) bursts at different sound levels. Representative raster plots from ACtx neurons show sound-evoked spiking in wild-type control mice, no sound-evoked activity in the ABR− cohort (Fig. 4a, middle) and robust spiking in ABR+ mice, but with higher thresholds (Fig. 4a, bottom, b, KStest *p* = 3.5e−116) and longer onset latencies than wild-type control mice (Fig. 4b, Kruskal–Wallis test *χ*^2^(1) = 64.19, *p* = 1.1e−15).

Since peripheral recovery after sAAV-*Tmc1* gene therapy was more prominent at the low frequency end of the cochlea (Fig. 2), we tested ACtx responses to pure tones rather than broadband stimuli. We presented mice with 361 frequency-level sound combinations and measured their frequency response areas (Fig. 4c). ABR− mice lacked tone-evoked responses for all frequencies and sound intensities tested. However, we found that frequency tuning bandwidth in ACtx recordings of ABR+ mice were similar to recordings from normally hearing control mice (Fig. 4d, Kruskal–Wallis test *χ*^2^(1) = 1.59, *p* = 0.2), but were biased towards higher thresholds (Fig. 4c, d, Kruskal–Wallis test *χ*^2^(1) = 199.95, *p* = 2.1e−45) and lower best frequencies (Fig. 4d, Kruskal–Wallis test *χ*^2^(1) = 162.55, *p* = 3.1e−37).

Neuroimaging and behavioral studies have shown enhanced visual activation of auditory cortex in deaf subjects, suggesting that congenital loss of one modality can lead to cross-modal recruitment of the deprived cortical region^26–28^. To investigate changes in the strength, speed and selectivity of visual processing in the auditory cortex of control, ABR− and ABR+ mice, we also presented drifting visual gratings of varying spatial frequency during the electrophysiology recording sessions (Fig. 4e, left). Among the small percentage of visually-driven neurons in the auditory cortex, we found that response latencies were significantly shorter in *Tmc1*^*∆/∆*^ mice that showed no evidence of cochlear recovery (ABR- mice; Kruskal–Wallis test *χ*^2^(2) = 12.6, pairwise comparisons p(ABR+, ABR−) = 1.2e−03 *p*(control, ABR−) = 2.9e−02), but found no difference between control and ABR+ mice, (Fig. 4f, g pairwise comparisons *p*(control, ABR+) = 0.16). These findings suggest that successful sAAV-*Tmc1* gene therapy can forestall cross-modal short latency visual recruitment of auditory cortex that occurs in deaf mice.

In summary, we found that improvements in sound-evoked responses in auditory cortex, although incomplete, were frequency specific with bandwidths similar to ACtx neurons of wild-type mice. Together, our recordings from primary auditory cortex show that central function follows peripheral recovery due to sAAV-*Tmc1* gene therapy.

Behavioral recovery of auditory function was tested by measuring acoustic startle responses in *Tmc1*^*∆/∆*^ mice injected with sAAV-*Tmc1*. We found that all of 11 P1-P2-injected mice had significantly improved startle responses compared to uninjected *Tmc1*^*∆/∆*^ mice (Fig. 4h), suggesting that even though ABR and DPOAE thresholds remained elevated, the recovery was sufficient to drive auditory behavior.

### Gene therapy restores vestibular function in Tmc1/2-null mice

**Fig. 5:**
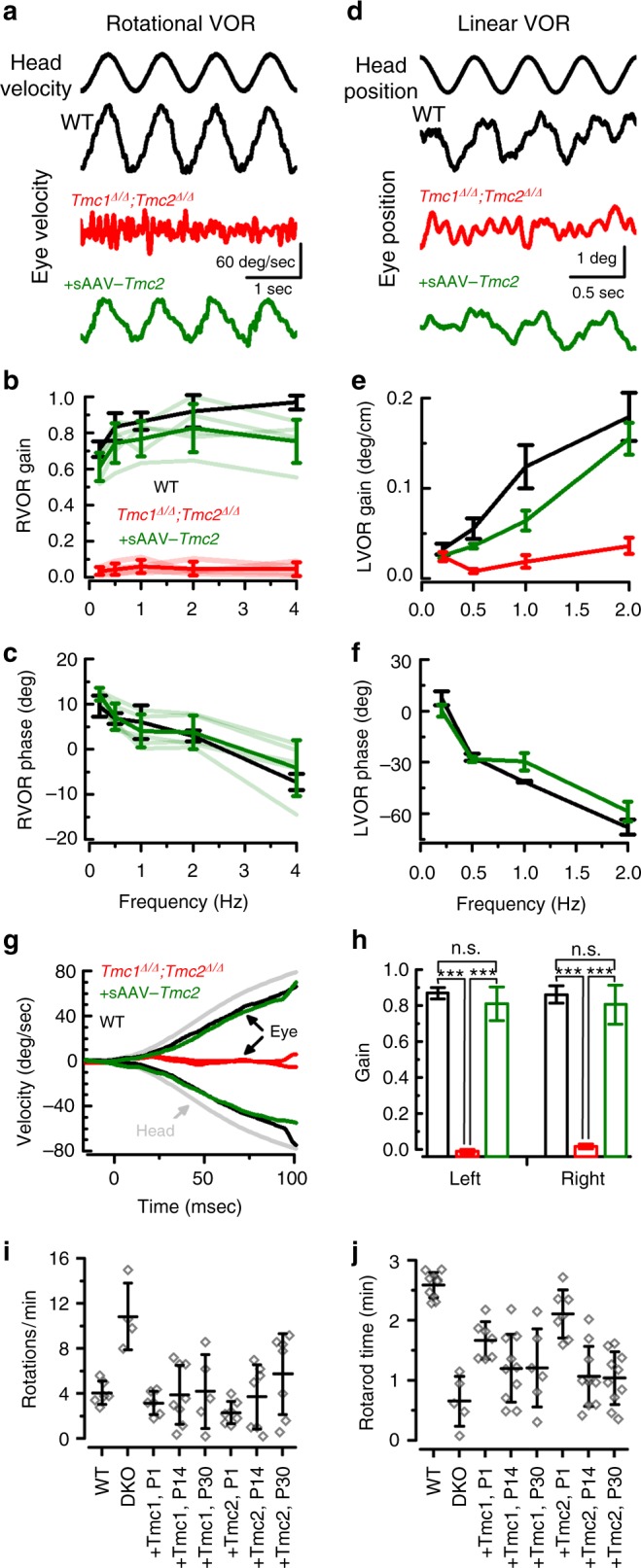
Exogenous *Tmc1* or *Tmc2* restores vestibular function in *Tmc1*^*∆/∆*^*;Tmc2*^*∆/∆*^ mice. **a** Rotational vestibular ocular reflexes (RVOR) to sinusoidal head rotations with representative eye velocity responses to 1 Hz head rotation for a wild-type mouse (black), a *Tmc1*^*∆/∆*^*;Tmc2*^*∆/∆*^ mouse (red), an sAAV-*Tmc2*-injected mouse (green). **b** Mean ± S.D. RVOR gains and **c** phases for WT group (*n* *=* 5, black), uninjected *Tmc* mutant mice (*n* = 4, red), and sAAV-*Tmc2*-treated group (*n* = 5, green). **d** Linear vestibular ocular reflexes (LVORs) to sinusoidal head translations with representative eye position measurements to 2 Hz head translations for a wild-type mouse (black), a *Tmc1*^*∆/∆*^*;Tmc2*^*∆/∆*^ mouse (red), an sAAV-*Tmc2*-injected mouse (green). **e** Mean ± S.D. gains and **f** phases of the LVORs are shown for uninjected *Tmc* mutant group (*n* = 4, red), the treated group (*n* = 5, green) and the WT group (*n* = 5, black). **g** Eye velocity response curves due to a high acceleration and high frequency step rotation. **h** Mean gains ± S.D. for eye movements during the first 100 ms, as shown in **g**. For **a**–**g**, positive head movements is for rightward head rotation and positive eye movement is for eye movement toward the left ear. Eye movements in injected and wild-type groups were compensatory to head rotation. Stars indicate statistical significance (*t*-test). **i** Mean ± S.D. number of full body rotations observed during 5 min in a 42-cm-wide arena in 6-week-old control *Tmc1*^*+/+*^*;Tmc1*^*+/+*^ mice, *Tmc1*^*∆/∆*^*; Tmc2*^*∆/∆*^ mice, and *Tmc1*^*∆/∆*^*; Tmc2*^*∆/∆*^ mice injected at either P1, P14 or P30 with sAAV-*Tmc1* or sAAV-*Tmc2*. Significant recovery *P* < 0.001 was observed between the uninjected and injected mice. Number of mice test for each of eight conditions were: 6, 4, 6, 8, 5, 7, 7, 7, respectively. Statistical analysis by one-way ANOVA. See Supplementary Movies 1-3. **j** Mean ± S.D. time spend on rotarod device recorded at 6 weeks of age in control *Tmc1*^*+/+*^*;Tmc1*^*+/+*^ mice, *Tmc1*^*∆/∆*^*; Tmc2*^*∆/∆*^ mice, and *Tmc1*^*∆/∆*^*; Tmc2*^*∆/∆*^ mice injected at either P1, P14 or P30 with sAAV-*Tmc1* or sAAV-*Tmc2*. Number of animals test for each of eight conditions were: 10, 5, 7, 10, 6, 7, 10, 10, respectively

Cochlear perilymph is continuous with perilymph that bathes vestibular organs, hence, gene therapy reagents injected into the cochlea are distributed throughout the inner ear perilymphatic spaces and transduce both auditory and vestibular hair cells^12,17^. Since both *Tmc1* and *Tmc2* are expressed in vestibular hair cells and *Tmc1*^*∆/∆*^*;Tmc2*^*∆/∆*^ mice suffer profound vestibular dysfunction^22^, we investigated whether *Tmc* gene therapy might improve vestibular function. We focused on *Tmc2* because it is expressed in vestibular organs at neonatal and mature stages^22^ and thus generated AAV2/Anc80L65*-Cmv-Tmc2-WPRE* vectors (sAAV-*Tmc2*). Following RWM injection of 1 µL sAAV-*Tmc2*, we measured rotational vestibular ocular reflexes (RVOR) to sinusoidal head rotations at 8 weeks of age. Representative eye velocity responses to 1 Hz head rotation for a *Tmc1*^*∆/∆*^*;Tmc2*^*∆/∆*^ mouse, an sAAV-*Tmc2*-injected mouse and a wild-type mouse are shown in Fig. 5a. While the *Tmc1*^*∆/∆*^*;Tmc2*^*∆/∆*^ mouse exhibited virtually no eye movement responses to head rotation, the treated mouse exhibited compensatory RVORs that were comparable to the RVORs of the WT mouse. Mean RVOR gains (Fig. 5b) and phases (Fig. 5c) for 14 mice are summarized for the three groups of mice. While the WT group (*n* *=* 5) exhibited high gain compensatory RVORs at all frequencies tested, the *Tmc* mutant mice (*n* = 4) exhibited virtually zero gain responses at the same frequencies and thus displayed little difference from responses of a dead mouse. However, the sAAV-*Tmc2*-treated group (*n* = 5) exhibited robust compensatory RVORs at these frequencies. Their phases were not statistically different from those of the WT mice. Although their gains were about 86% of that of the WT group, the differences between experimental mice and wild-type mice did not reach statistical significance at the tested frequencies (*t*-test: 0.2 Hz: *p* *=* 0.305; 0.5 Hz: *p* *=* 0.524; 1 Hz: *p* *=* 0.401; 2 Hz: *p* *=* 0.573; 4 Hz: *p* *=* 0.127).

To assess the effects of sAAV-*Tmc2* gene therapy on the otolith system (utricle and saccule), we measured linear vestibular ocular reflexes (LVORs) to sinusoidal head translations. Figure 5d shows representative eye position responses to 2 Hz head translations for a WT mouse (black), a *Tmc1*^*∆/∆*^*;Tmc2*^*∆/∆*^ mouse (red), and a sAAV-*Tmc2*-treated mouse (green). While the *Tmc* mutant mouse exhibited virtually no eye movement responses to head translation, the treated mouse exhibited compensatory LVORs that were similar to the responses of the WT mouse. Mean gains (Fig. 5e) and phases (Fig. 5f) of the LVORs are shown for the three groups of mice. In contrast to the *Tmc* mutant group (*n* *=* 4), which exhibited virtually zero gain responses at these frequencies, the treated group (*n* *=* 5) exhibited compensatory responses. Phases of the injected mice were nearly the same as the WT group (*n* = 5). Gains were not significantly different from those of the WT group at all frequencies tested (*t*-test: 0.2 Hz, *p* *=* 0.3; 0.5 Hz, *p* *=* 0.14; 1 Hz, *p* = 0.053; 2 Hz: *p* = 0.762).

In order to reliably track eye movements, all VOR measurements were performed in ambient light conditions. To rule out the possibility that visual input drove the eye movement responses in Fig. 5a–f, we employed not only high frequency sinusoidal head rotations (e.g., 2 and 4 Hz), but also high acceleration and high frequency step rotations. Because the onset of visually-evoked eye movements requires at least 80 ms, the first 80 ms of the eye movement responses are primarily driven by vestibular inputs, i.e., open-loop responses without visual feedback. Figure 5g-h show that the sAAV-*Tmc2*-treated group exhibited nearly identical response latencies to rapid head rotations as the WT group (*p* *=* 0.64, *t*-test). Thus, the sAAV-*Tmc2* gene therapy in *Tmc1*^*∆/∆*^*;Tmc2*^*∆/∆*^ mice promoted VOR responses at rates that were too rapid to be explained by visual compensation, supporting the conclusion for robust vestibular recovery.

These results not only confirmed a previous report of absence of RVOR in *Tmc1*^*∆/∆*^*;Tmc2*^*∆/∆*^ mice^22^, but also extend that finding to include loss of LVORs, suggesting that *Tmc1/2* disruption affected both the semicircular canals and the otolith organs. Importantly, the data demonstrate that sAAV-*Tmc2* gene therapy restores VOR responses to near wild-type levels.

To assess vestibular recovery at the behavioral level, we recorded open-field behavior over the course of five minutes in six-week-old control WT mice, *Tmc1*^*∆/∆*^*; Tmc2*^*∆/∆*^ mice, and *Tmc1*^*∆/∆*^*; Tmc2*^*∆/∆*^ mice injected at P1, P14, or P30 with sAAV-*Tmc1* or sAAV-*Tmc2*. Figure 5i demonstrates that *Tmc1*^*∆/∆*^*; Tmc2*^*∆/∆*^ mice, which have profound vestibular dysfunction, circle 10–12 times each minute (Supplementary Movies 1-3), significantly more than wild-type mice (*p* = 7.6E−4, ANOVA). *Tmc1*^*∆/∆*^*; Tmc2*^*∆/∆*^ mice injected with sAAV-*Tmc1* or sAAV-*Tmc2* at all three time points displayed significantly less circling behavior (*p* = 4.4E−4, ANOVA).

To assess balance behavior on a more challenging task, we recorded rotarod performance at six weeks of age in the same cohorts of mice. While wild-type mice maintained balance on a rotating rod for 2–3 min on average, *Tmc1*^*∆/∆*^*; Tmc2*^*∆/∆*^ mice typically fell off the device within 30–40 s (Fig. 5j). Injection of sAAV-*Tmc1* or sAAV-*Tmc2* at early postnatal stages (P1) yielded the best improvement in rotarod performance, with mice able to maintain balance for 1.5–3 min. Injection at later stages also yielded improvement in balance behavior, though to a lesser extent, perhaps due to reduced viral transduction in vestibular hair cells when injected at later postnatal stages (Supplementary Fig. 6).

### Tmc1 gene therapy improves secondary outcomes

**Fig. 6:**
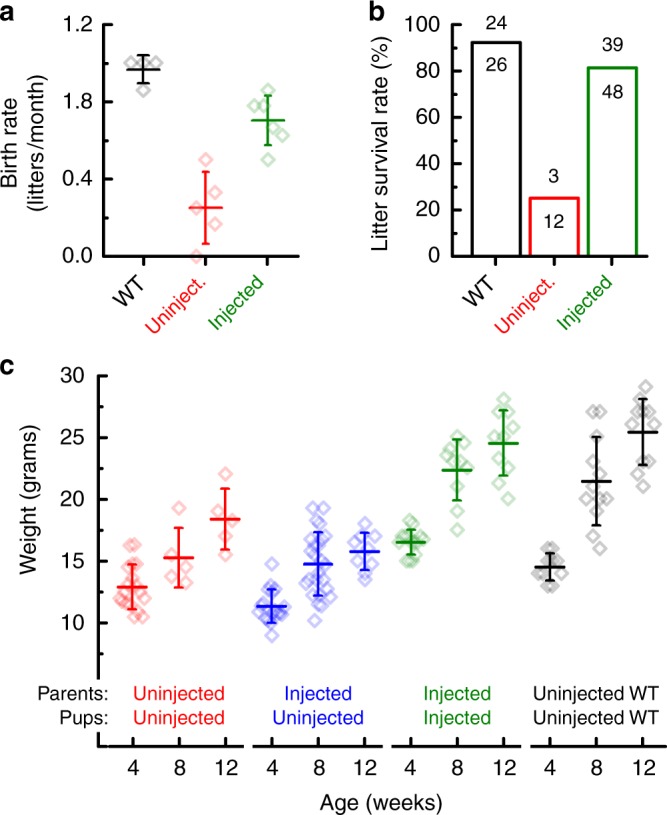
sAAV-*Tmc1* improves breeding, survival and growth in *Tmc1*^*∆/∆*^*;Tmc2*^*∆/∆*^ mice. **a** Mean ± S.D. birth rate for litters produced by four wild-type C57BL/6 breeding pairs (black), five uninjected *Tmc1*^*∆/∆*^*;Tmc2*^*∆/∆*^ breeding pairs (red) and six *Tmc1*^*∆/∆*^*;Tmc2*^*∆/∆*^ breeding pairs injected with sAAV-*Tmc1* (green). Birth rate was calculated for each breeding pair based on number of litters divided by duration mated. **b** Litter survival rates wild-type (black), uninjected *Tmc1*^*∆/∆*^*;Tmc2*^*∆/∆*^ mice (red), and *Tmc1*^*∆/∆*^*;Tmc2*^*∆/∆*^ parents injected with sAAV-*Tmc1* (green). Number of litters that survived to wean age (P21) over number produced at P0 are shown above each bar. Litters were classified as non-survival if none of the pups from the litter survived to P21. **c** Mean ± S.D. mouse weights were taken for uninjected *Tmc1*^*∆/∆*^*;Tmc2*^*∆/∆*^ mice (red), uninjected *Tmc1*^*∆/∆*^*;Tmc2*^*∆/∆*^ mice whose *Tmc1*^*∆/∆*^*;Tmc2*^*∆/∆*^ parents were injected with sAAV-*Tmc1* (blue), *Tmc1*^*∆/∆*^*;Tmc2*^*∆/∆*^ mice injected on P1 with sAAV-*Tmc1* whose parents were also *Tmc1*^*∆/∆*^*;Tmc2*^*∆/∆*^ mice injected on P1 with sAAV-*Tmc1* (green), and C57BL6/J controls for comparison (black) at 4, 8, and 12 weeks of age as indicated

*Tmc1*^*∆/∆*^*;Tmc2*^*∆/∆*^ mice suffer profound hearing and balance dysfunction, consequently, *Tmc1*^*∆/∆*^*;Tmc2*^*∆/∆*^ mice do not breed well. When they do breed, survival of *Tmc1*^*∆/∆*^*;Tmc2*^*∆/∆*^ offspring to *Tmc1*^*∆/∆*^*;Tmc2*^*∆/∆*^ parents is reduced and those that do survive have stunted growth relative to wild-type C57BL/6 mice. In the course of this study we discovered that *Tmc1*^*∆/∆*^*;Tmc2*^*∆/∆*^ mice injected with of sAAV-*Tmc1* were better breeders (Fig. 6a). During a 6–7 month mating period, four wild-type C57BL/6 breeding pairs produced 6–7 litters each, or ~1 litter/month. During a 6–12 month breeding period, five *Tmc1*^*∆/∆*^*;Tmc2*^*∆/∆*^ breeding pairs produced an average of three litters/breeding pair or an average of 1 litter every four months. *Tmc1*^*∆/∆*^*;Tmc2*^*∆/∆*^ breeding pairs in which both parents were injected with sAAV-*Tmc1*, produced 8–9 litters during the same 6–12 month time frame. In addition to the significant (*p* = 0.001, *t*-test) increase in the number of litters, we also found that there was an increase in survival rate among the litters born to sAAV-*Tmc1*-injected *Tmc1*^*∆/∆*^*;Tmc2*^*∆/∆*^ breeding pairs. On average 92% of litters from WT parents survive to their typical wean date of P21, whereas only 22% of litters born to *Tmc1*^*∆/∆*^*;Tmc2*^*∆/∆*^ parents survived to weaning age. In contrast, 81% of litters born to *Tmc1*^*∆/∆*^*;Tmc2*^*∆/∆*^ mice injected with sAAV-*Tmc1* survived until weaning (Fig. 6b).

We also tracked weight and growth of pups at four, eight and 12 weeks of age among four cohorts of mice: (1) WT C57B/L6, (2) *Tmc1*^*∆/∆*^*;Tmc2*^*∆/∆*^ pups born to *Tmc1*^*∆/∆*^*;Tmc2*^*∆/∆*^ parents, (3) *Tmc1*^*∆/∆*^*;Tmc2*^*∆/∆*^ pups born to sAAV-*Tmc1*-injected parents, and (4) of sAAV-*Tmc1*-injected pups born to sAAV-*Tmc1*-injected parents. WT mice doubled their weight from ~12 to 24 g between 4 and 12 weeks of age (Fig. 6c). *Tmc1*^*∆/∆*^*;Tmc2*^*∆/∆*^ pups born to *Tmc1*^*∆/∆*^*;Tmc2*^*∆/∆*^ parents had similar weight to WT mice at 4 weeks of age, but failed to thrive, growing only ~50% during the following 8 weeks. *Tmc1*^*∆/∆*^*;Tmc2*^*∆/∆*^ pups born to sAAV-*Tmc1*-injected parents did not fare any better and their growth remained stunted over the same time frame. However, sAAV-*Tmc1*-injected pups born to sAAV-*Tmc1*-injected parents showed a dramatic improvement in growth with weights that were indistinguishable from those of WT mice at all three time points tested. These data indicate that recovery of hearing and balance function has a significant impact on secondary measures including, breeding, survival and growth in mouse models of genetic inner ear disorder.

## Discussion

Here we demonstrate significant improvement in auditory and vestibular function using next generation gene therapy in a mouse model of the human recessive deafness DFNB7/11. The difference over prior gene therapy outcomes for recessive *Tmc1* mutations^12^ represents a substantial qualitative improvement in outcomes. Quantitative comparison between studies that use different vectors, methods of vector generation and titration, injection and outcome assays is challenging. For the current study, the viral titers used were 5 to 10-fold higher than the titer used in a prior study^12^, yet we found qualitatively similar results using the AAV2/1 capsid to deliver *Tmc1*: recovery of IHC function and partial recovery of ABR thresholds, but minimal recovery of OHC function and DPOAEs, the latter likely due to lack of viral transduction in outer hair cells. Because OHCs amplify and tune the sound stimulus and their dysfunction or loss causes disruption of cochlear amplification and hence reduced auditory sensitivity, we generated sAAV vectors with *Cmv* promoters to drive exogenous *Tmc1* expression in both IHCs and OHCs. In normal hearing mice, both IHCs and OHCs express WT *Tmc1*, thus, *Tmc1* gene replacement in both cell types is required to overcome the consequences of recessive loss-of-function mutations in *Tmc1*. Thirty-five *TMC1* mutations have been identified in humans^29^, including several with moderate-to-severe hearing loss phenotypes^10,11^, some of which may be amenable to *TMC1* gene therapy. In our mouse model of recessive *TMC1* mutations, we found enhanced survival of IHCs and OHCs in sAAV-*Tmc1* injected cochleas, robust uptake of the transduction channel permeable dye, FM1-43, and wild-type mechanosensory transduction currents in large numbers of sAAV-exposed IHCs and OHCs.

The level and extent of cellular recovery suggested that organ function was likely restored as well. Indeed, the average improvement in ABR thresholds in the sAAV-*Tmc1*-injected mice was ~30 dB (at 8 kHz) better than average thresholds obtained using AAV2/1 vectors^12^. In some of the best examples, DPOAE and ABR responses indicated auditory thresholds as low as 35 dB in sAAV-injected mice, which represents an improvement of auditory thresholds by 50 dB. In other words, in the best cases, sAAV-injected mice were able to hear sounds with intensities that were 100,000-fold softer than AAV2/1 injected mice. Furthermore, thresholds in some cases were close to those of wild-type mice, ~25 dB. Importantly, the best recovery of auditory thresholds in sAAV-*Tmc1*-injected mice was at the low frequency end of the mouse auditory spectrum. In humans, the frequency range of spoken language is at the low to mid frequency end of the auditory spectrum. ABR thresholds in injected mice were also in the sound intensity range of the conversational human voice: 30–60 dB. Thus, if DFNB7/11 humans were to recover similar levels of auditory function as the *Tmc1*-injected mice, we suggest perception of human speech may be feasible. Strategies for recovery of auditory function at the high frequency end of the cochlea will require further investigation.

Since auditory perception requires complex processing of auditory signals in the ascending auditory pathways, we asked whether restoration of auditory function in the periphery was sufficient to drive neural responses in auditory cortex. Prior studies have shown that eliminating ~95% of cochlear afferent neurons renders the ABR absent or grossly abnormal, yet cortical neurons can retain relatively normal responses^30^. In *Tmc1*-null mice without ABR recovery, where cochlear dysfunction is virtually complete, central compensatory plasticity did not restore cortical processing and therefore central functioning followed the status of the periphery. In addition, recordings from auditory cortex of *Tmc1*^*∆/∆*^ mice without ABR recovery revealed abnormally elevated spontaneous firing rates and abnormally short latency neural responses to visual stimuli.

In sAAV-*Tmc1*-injected *Tmc1*^*∆/∆*^ mice with improved ABR thresholds, we found stimulus-evoked responses in auditory cortical neurons. The tuning curves of the neurons had bandwidths similar to those of wild-type mice. While the thresholds and frequency ranges were elevated and centered around 8 kHz, the data indicated robust auditory-evoked responses in auditory cortex. This result has several implications: (1) Central auditory processing was documented by action potential recordings from auditory cortex, a more sensitive assay than the far-field electrical potentials measured by ABR. (2) Local delivery of *Tmc1* gene therapy to the cochlea was sufficient to drive neural responses in ascending auditory pathways. (3) Thus, neuronal function in auditory cortex does not require *Tmc1* expression locally within the cortex. Since some genes that cause deafness when mutated are expressed in both the cochlea and ascending auditory pathways^24,25^, restoration of function in auditory cortex following peripheral injection of sAAV-*Tmc1* was not assured. (4) The neuronal responses in auditory cortex were correlated with recovery of ABR responses. This suggests that for *Tmc1* gene therapy, the ABR, a less invasive measure, may be a good proxy for recovery of neural activity in higher auditory pathways, at least at early developmental stages. Whether normal function of auditory cortex can be recovered following gene therapy in the peripheral auditory system when introduced at mature stages remains to be determined. Further enhancement of cortical responses may also be achieved by injection of both ears and will require further investigation.

We also found significant recovery of vestibular function in *Tmc1/Tmc2* double mutant mice injected with sAAV-*Tmc2* vectors. Recovery of vestibular ocular reflexes in response to rotational or linear head movements suggested recovery of function in semi-circular canal and otolith organs, respectively. Behavioral responses in open field and rotarod tasks also showed improved balance for all conditions examined, including injection of sAAV-*Tmc1* or sAAV-*Tmc2*, as well as injection at mature stages, P30 (the latest time point tested). Gene therapy recovery of balance at mature stages has not been reported previously but raises several important points: (1) vestibular hair cells remain viable at mature stages, (2) sAAV-*Tmc* can transduce more mature vestibular hair cells, (3) recovery of cellular function and vestibular behavior is possible at later stages. This also highlights an important difference between auditory and vestibular hair cells and suggests that strategies for targeting mature auditory hair cells may benefit from a better understanding of viral transduction in vestibular hair cells.

The recovery of balance behavior and VORs to near wild-type levels in injected *Tmc1/Tmc2* double mutant mice provides compelling proof-of-concept evidence supporting development of human gene therapy for genetic vestibular dysfunction. Although genetic balance dysfunction has been poorly characterized, balance disorders and falls are common in the elderly. We previously demonstrated that sAAVs can transduce human vestibular hair cells in vitro^18^. Since the perilymphatic solutions of the human cochlea are continuous with those of the vestibular organs, we suspect that RWM injection may be a suitable delivery route for targeting human vestibular hair cells in vivo. As such, we propose that vestibular gene therapy, perhaps in both ears, and even at mature stages, may provide a viable future treatment option for balance disorders, and thus provide a therapeutic intervention for a significant unmet medical need.

Mice that carry *Tmc1/Tmc2* mutations are deaf and suffer balance dysfunction, consequently, *Tmc1/Tmc2*-null animals do not breed well, offspring have lower survival rates and stunted growth. We found that *Tmc1/Tmc2*-null breeding pairs injected with sAAV-*Tmc1* produced litters at a higher rate, ~8 litters/year. Litters born to sAAV-*Tmc1*-injected parents had significantly higher survival rate, with ~80% of litters surviving to wean age, P21. Furthermore, *Tmc1/Tmc2*-null pups that were injected with sAAV-*Tmc1* unilaterally and born to sAAV-*Tmc1*-injected parents, had weights and growths that were indistinguishable from age-matched wild-type control pups. Thus, using measures of breeding success, survival rates and growth rates, the data suggest that, in addition to enhanced auditory and vestibular function, *Tmc* gene therapy restores secondary measures in mouse models of genetic inner ear dysfunction. While normal auditory and balance function have been long recognized as contributing factors for quality of life in humans, here for the first time, we demonstrate that gene therapy restoration of hearing and balance enhance several secondary outcomes in a mouse model of human inner ear dysfunction.

Given the extent of the functional recovery quantified here using multiple physiological and behavioral assays, we suggest that *Tmc* gene replacement therapy for recessive genetic inner ear dysfunction may be well suited for further development and translation to the clinic. Future studies that extend the recovery to the high frequency end of the auditory spectrum, identify strategies for recovery at later stages of intervention and explore the range of human mutations amenable to therapeutic intervention, may further enhance the outcome of inner ear gene therapies.

## Methods

### Study design

The aim of this study was to use the Anc80L65 capsid for delivery and expression of exogenous *Tmc1ex1* and *Tmc2* in hair cells of the mouse cochlea and vestibular organs and to evaluate the ability of these vectors to restore auditory and vestibular function in mouse models of genetic inner ear disorders. Anc80L65 vectors containing the coding sequence for *eGFP*, *Tmc1*, or *Tmc2* were injected in vivo through the round window membrane, and the outcomes were evaluated using FM1-43 uptake, single-cell recording, measurement of ABRs, DPOAEs, confocal microscopy, and tonotopic mapping of whole cochleas, acoustic startle reflexes, multi-unit auditory cortex recordings, rotarod and open field analysis, vestibular ocular reflexes, and secondary indicators of functional recovery. Left ears were injected postnatally as indicated and later time points of injection were used to assess the effective therapeutic window. Each experiment was replicated as indicated by *n* values in the figure legends. All experiments with mice and viral vectors were approved by the Institutional Animal Care and Use Committee (protocols #18-01-3610R and #17-03-3396R) and the Institutional Biosafety Committee (protocol #IBC-RN00000447-1) at Boston Children’s Hospital.

### Mice

Wild-type control mice were C57BL/6J (Jackson Laboratories) and mice that carried mutant alleles of *Tmc1* and *Tmc2* were on a C57BL/6J background^22^. Three genotypes of *Tmc* mutant mice were used: *Tmc1*^*Δ/Δ*^*; Tmc2*^*+/+*^*, Tmc1*^*Δ/Δ*^*;Tmc2*^*Δ/+*^, and *Tmc1*^*Δ/Δ*^*;Tmc2*^*Δ/Δ*^. Mice ages P0-P1, P4, P7, and P14 were used for in vivo delivery of viral vectors according to protocols approved by the Institutional Animal Care and Use Committee (protocols #18-01-3610R and #17-03-3396R) at Boston Children’s Hospital.

### Tissue preparation

Temporal bones were harvested from mice at P7, P28, or 12 weeks after birth. For tissues harvested at P7, pups were euthanized by rapid decapitation and temporal bones were dissected in Minimum Essential Medium (Invitrogen) supplemented with 10 mM HEPES, 0.05 mg/mL ampicillin, and 0.01 mg/mL ciprofloxacin at pH 7.40. The membranous labyrinth was isolated under a dissection scope, Reissner’s membrane was peeled back, and the tectorial membrane and stria vascularis were mechanically removed. Organ of Corti cultures were pinned flatly beneath a pair of thin glass fibers adhered at one end with Sylgard to an 18-mm round glass coverslip. The tissue was placed in culture and used acutely for electrophysiological studies. For mice at P28 or older, temporal bones were harvested after euthanizing the animal with inhaled CO_2_, and cochlear half-turns were isolated under a dissection scope via removal of lateral wall tissue and Reissner’s membrane with breakable blades (Fine Science Tools) and mechanical removal of tectorial membrane for generation of whole mount free-floating tissues.

### Vector production

Anc80L65 vectors carrying the coding sequence for *eGFP*, mouse *Tmc1*, or mouse *Tmc2* driven by a cytomegalovirus (*Cmv*) promoter were generated using a helper virus free system and a double transfection method^31^. AAV2/1-*Cmv-Tmc1-WPRE*, AAV2/Anc80-*Cmv*-*eGFP-WPRE*, AAV2/Anc80-*Cmv-Tmc1ex1-WPRE*, and AAV2/Anc80-*Cmv*-*Tmc2-WPRE* were produced by the Viral Core at Boston Children’s Hospital at titers of 8.1E+14 gc/mL, 1.4E+13 gc/mL, 1.4E+14 gc/mL, and 1.6E+14 gc/mL, respectively. Titers were calculated by qPCR with GFP primers (GFP-F: AGAACGGCATCAAGGTGAAC; GFP-R: GAACTCCAGCAGGACCATGT), or ITR primers (LITR-F: GACCTTTGGTCGCCCGGCCT; LITR-R: GAGTTGGCCACTCCCTCTCTGC). All four vectors were purified using an iodixanol step gradient followed by ion exchange chromatography. Virus aliquots were stored at −80 °C and thawed just prior to use for in vivo injections.

### In vivo injection of viral vectors

Mouse pups were injected via the round window membrane (RWM) technique using beveled glass microinjection pipettes on postnatal day (P) between P0 and P30 as indicated. Pipettes were pulled from capillary glass on a P-2000 pipette puller (Sutter Instruments). Pups were anesthetized by rapid induction of hypothermia for 2–4 min on ice water until loss of consciousness, and this state was maintained on a cooling platform for 10–15 min during the surgery. The surgical site was disinfected by scrubbing with Betadine and wiping with 70% Ethanol. A post-auricular incision was made to expose the transparent otic bulla, and the RWM was penetrated by the tip of the micropipette. Approximately 1 μL of virus was slowly introduced unilaterally into the left ear. The skin incision was closed using a 6-0 monofilament suture (Ethicon). EMLA cream (lidocaine 2.5% and prilocaine 2.5%) was applied externally for analgesia using sterile swabs to cover the surgical site (left mastoid prominence). Body temperature was maintained on a 37 °C warming pad for 30–60 min after surgery and before reintroduction into parental cage.

### Hair cell electrophysiology

Organotypic cochlear cultures were bathed in standard artificial perilymph containing 137 mM NaCl, 0.7 mM NaH_2_PO_4_, 5.8 mM KCl, 1.3 mM CaCl_2_, 0.9 mM MgCl_2_, 10 mM HEPES, and 5.6 mM d-glucose. Vitamins (1:50) and amino acids (1:100) were added to the solution from concentrates (Invitrogen), and NaOH was used to adjust the final pH to 7.40 (310 mosmol/kg). Recording pipettes (3–5 megohms) were pulled from R6 capillary glass (King Precision Glass) and filled with intracellular recording solution containing 135 mM CsCl, 5 mM HEPES, 5 mM EGTA, 2.5 mM MgCl_2_, 2.5 mM Na_2_-adenosine triphosphate, and 0.1 mM CaCl_2_, where CsOH was used to adjust the final pH to 7.40 (285 mosmol/kg). Whole-cell, tight-seal voltage-clamp recordings were done at −84 mV at room temperature (22–24 °C) using an Axopatch 200B amplifier (Molecular Devices). Hair bundles were deflected with a stiff glass probe fabricated from capillary glass with a fire polisher (MF-200, World Precision Instruments) for creating a rounded probe tip of ~3–5 μm in diameter^32^. Probes were mounted on a PICMA Chip piezo actuator (Physik Instrument, Karlsruhe, Germany) and driven by an LVPZT amplifier (E-500.00, Physik Instrumente, Karlsruhe, Germany). Sensory transduction currents were filtered at 10 kHz with a low-pass Bessel filter and digitized at ≥20 kHz with a 16-bit acquisition board (Digidata 1440A) and pCLAMP 10 software (Molecular Devices). Bundle deflections were monitored via video microscopy during the recording to ensure linear probe motion and coupling of the probe to the hair bundle during mechanical steps. Data were stored for offline analysis using OriginPro 2016 (OriginLab).

### FM1-43 labeling

FM1-43 dye loading experiments were performed in vitro^20,21,33^. Coverslips with adherent cochlear cultures were placed under an upright microscope (Zeiss Axioscope FS Plus) on a glass-bottomed chamber. Five micrometer FM1-43 (Invitrogen) diluted in artificial perilymph was applied for 10 s and the tissue was washed three times in artificial perilymph to remove dye from the outer leaflet of the cell membrane. After 5 min, intracellular FM1-43 was imaged using an FM1-43 filter set and an epifluorescence light source with a 63× water immersion objective.

### Confocal Immunofluorescence

Temporal bones dissected for immunohistochemistry were immersion fixed for 1 h at room temperature with 4% paraformaldehyde diluted in PBS after mechanical disruption of the oval and round window membranes and removal of an apical chip for fixative access throughout the cochlea. Post fixation, temporal bones were decalcified in 120 mM EDTA for 24 h or 1.5 days for P28 or 12-week-old tissues, respectively, before microdissection and isolation of either the organ of Corti or utricle. The tissue was then rinsed in PBS and permeabilized in 0.01% Triton X-100 for 1 h. For hair cell counts or calculation of transduction efficiency, tissue was blocked in 2.5% normal donkey serum and 2.5% bovine serum albumin diluted in PBS (blocking solution) for 1 h and subsequently stained with a rabbit anti-Myosin VIIa primary antibody (Proteus Biosciences, Product #: 25-6790, 1:500 dilution in blocking solution) at 4 °C overnight. A secondary antibody cocktail consisting of a mixture of donkey anti-rabbit antibody conjugated to AlexaFluor555 (Life Technologies, 1:200 dilution) and AlexaFluor647-phalloidin (Molecular Probes, 1:200 dilution) as a counterstain to label filamentous actin was applied for 4–5 h. Localization of exogenously expressed eGFP protein was performed without antibody enhancement. Samples were mounted on glass coverslips with Vectashield mounting medium (Vector Laboratories), and imaged at 10×-63× magnification using a Zeiss LSM800 confocal microscope. Three-dimensional projection images were generated from *Z*-stacks using ZenBlue (Zeiss).

### Auditory brainstem responses

ABR recordings were conducted from mice anesthetized via IP injection (0.1 mL/10 g–body weight) with 1 mL of ketamine (50 mg/mL) and 0.75 mL of xylazine (20 mg/mL) diluted into 8.25 ml of 0.9% saline^12^ at time points indicated in the text. ABR experiments were performed at 32 °C in a sound-proof chamber. To test hearing function, mice were presented pure tone stimuli of 5.6, 8, 11.3, 16, 22.6, or 32 kHz at sound pressure levels between 10 and 115 dB in 5 dB steps until a threshold intensity that evoked a reproducible ABR waveform with an identifiable Peak 1 was detected. Using an alternating polarity stimulus, 512 responses were collected and averaged for each sound pressure level. Waveforms with amplitude larger than 15 μV (peak-to-trough) were discarded by an artifact-reject function. Prior to the onset of ABR testing, the flap of skin and cartilage that typically obscures the entrance of the external auditory meatus was trimmed away with dissecting scissors, and sound pressure at the entrance of the ear canal was calibrated for each individual test subject at all stimulus frequencies. Acoustic stimuli were delivered directly to the studied ear through a custom probe tube speaker/microphone assembly (EPL PXI Systems) consisting of two electrostatic earphones (CUI Miniature Dynamics) to generate primary tones and a Knowles miniature microphone (Electret Condenser) to record ear-canal sound pressure. Sound stimuli consisted of 5-ms tone bursts (0.5 ms rise-fall with a cos^2^ onset, delivered at 40 Hz). ABR signals were collected using subcutaneous needle electrodes inserted at the pinna (active electrode), vertex (reference electrode), and rump (ground electrode). ABR potentials were amplified (10,000×), pass-filtered (0.3–10 kHz), and digitized using custom data acquisition software (LabVIEW) from the Eaton-Peabody Laboratories Cochlear Function Test Suite. Sound stimuli and electrode voltage were sampled at 40-μs intervals using a digital I-O board (National Instruments) and stored for offline analysis. Threshold was defined visually as the lowest decibel level at which peak I could be detected and reproduced with increasing sound intensities. ABR thresholds were averaged within each experimental group and used for statistical analysis. ABR and DPOAE measurements were performed by investigators blinded to the genotype.

### DPOAE measurement

DPOAE data were collected under the same conditions, and during the same recording sessions, as ABR data. Primary tones were produced at a frequency ratio of 1.2 (f2/f1) for the generation of DPOAEs at 2f1-f2, where the f2 level was 10 dB sound pressure level below f1 level for each f2/f1 pair. The f2 levels were swept in 10-dB steps from 20 to 80 dB. Waveform and spectral averaging were used at each level to increase the signal-to-noise ratio of the recorded ear-canal sound pressure. The amplitude of the DPOAE at 2f1-f2 was extracted from the averaged spectra, along with the noise floor at nearby points in the spectrum. Iso-response curves were interpolated from plots of DPOAE amplitude versus sound level. Threshold was defined as the f2 level required to produce DPOAEs above 0 dB.

### Auditory cortex recordings

All procedures were approved by the Animal Care and Use Committee at the Massachusetts Eye and Ear Infirmary and followed guidelines established by the National Institutes of Health for the care and use of laboratory animals. Behavior and awake extracellular recordings were performed at 7–10 weeks old on *C57BL/6J* and *Tmc1*-deficient mice injected at P1-2 with AAV2/Anc80L65-*Cmv-Tmc1ex1.WPRE*.

A titanium head plate was affixed to the skull with dental cement (C&B Metabond) three days before recording sessions began. The primary auditory cortex was first identified by anatomical markers (2.5 mm posterior to bregma lateral to the squamosal ridge) and through a coarse mapping of middle layer unit responses when possible. Before the first recording session, animals were briefly anesthetized with isoflurane (1.5% in oxygen) while a small craniotomy (0.5 × 1.0 mm, medial-lateral × rostral-caudal) was made along the caudal end of the right temporal ridge, 1 mm rostral to the lambdoid suture to expose A1. A small chamber was built around the craniotomy with UV-cured cement and filled with ointment. At the end of each recording session, the chamber was flushed, filled with fresh ointment, and sealed with UV-cured cement. The chamber was removed and rebuilt under isoflurane anesthesia before each subsequent recording session. On the day of recording, the head was immobilized by attaching the head plate to a rigid clamp (Altechna). A 16-channel silicon probe (NeuroNexus Technologies) was inserted orthogonal to the brain surface in A1. Raw neural signals were digitized at 32-bit, 24.4 kHz (RZ5 BioAmp Processor; Tucker-Davis Technologies) and stored in binary format for offline analysis. The signal was bandpass filtered at 300–3000 Hz with a second-order Butterworth filter and movement artifacts were minimized through common mode rejection.

Acoustic stimuli were generated with a 24-bit digital-to-analog converter (National Instruments model PXI-4461). Stimuli were presented via free-field electrostatic speakers (Tucker-Davis Technologies) facing the left (contralateral) ear. Free-field stimuli were calibrated before recording with a wideband ultrasonic acoustic sensor (Knowles Acoustics, model SPM0204UD5).

Visual stimuli were generated in Matlab using the Psychtoolbox extension and displayed on a monitor (Dell, 17 inch, 60 Hz refresh rate) placed 25 cm from the mouse. Drifting gratings were presented full screen for 1 s. Five spatial frequencies were presented (0.02, 0.04, 0.08, 0.16, and 0.32).

Broadband noise bursts (4–64 kHz, 0.1 s duration, 4 ms raised cosine onset/offset ramps) were presented at 0–90 dB SPL in 5 dB increments. Each noise level was presented 12 times. Threshold was defined as the lowest of at least two continuous stimulus levels for which the response to sound was significantly higher than the spontaneous activity.

Frequency response areas (FRAs) were measured with pseudorandomly presented tone pips (50 ms duration, 4 ms raised cosine onset/offset ramps, 0.5–1 s intertrial interval) of variable frequency (4–48 kHz in 0.2 octave increments) and level (0–90 dB SPL in 5 dB increments). Each tone pip was repeated three times and responses to each iteration were averaged. The start of the spike collection window was set to be the point when the firing rate began to consistently exceed the spontaneous rate by at least 3 SD. The offset of the spike collection window was the first bin after the response decreased to less than 5 SD above the spontaneous rate. Additional details on spike windowing and FRA boundary determination are described in Guo et al. (2012)^34^.

For both auditory and visual responses, latency to onset was defined as the time-point at which the post-stimulus firing rate exceeded spontaneous firing rate by 3.5 SDs. For auditory responses latency was measured 10 dB above threshold.

### Acoustic startle reflexes

Mice were tested for startle reflexes in response to broadband auditory stimulation at varying intensities. The animals were tested in a sound shielded startle booth (Kinder Scientific) and the force generated by foot movement was sensed by piezoelectric motion sensor fixed beneath an elevated platform. Mice were placed in a smaller sub chamber anchored to the topside of the platform, which restricted them from rearing on their limbs but freely permitted penetration of the sound stimulus. Sound stimuli were calibrated with the door closed using a sound pressure level meter (Allied Electronics) with the microphone mounted in the position normally occupied by the animal holder. The generation of sound stimuli and recording of force amplitude signals was performed by startle monitor software (Kinder Scientific). Broadband white noise was presented at a background level of 60 dB throughout the experiment and auditory test stimuli consisted of 50-ms broadband white noise pulses in 10-dB steps from 60 to 120 dB. Different intensities of the test stimulus were presented in a pseudo-random order at randomized inter trial intervals (ITI) that varied between 8 and 22 s, with no ITI repeated more than three times. Five repetitions were averaged for each of the intensities for one test subject and responses were normalized to the weight of the mouse tested. Startle response measurements were performed by investigators blinded to the genotype.

### Vestibular ocular reflexes (VOR)

Tests were performed on *Tmc1/2* double knockout and *Tmc1/2* double knockout injected on P1 with sAAV-*Tmc2* sent from the Holt Lab, and wild-type C57BL/6J mice. All procedures were carried out in accordance with NIH guidelines and approved by the Institutional Animal Care and Use Committee at the University of Mississippi Medical Center. All surgical procedures were performed aseptically^35^. Briefly, each mouse was implanted with a small head holder on the skull and was allowed seven days to recover before eye movement tests. Horizontal and vertical eye position signals were recorded using a video-based eye tracking system (ISCAN ETS-200, ISCAN, Burlington, MA). An infrared camera equipped with a zoom lens (Computar TV Zoom Lens, Computar Optics Group, Japan) was attached to the platform mounted on a servo-controlled rotator/sled (Neurokinetic, Pittsburgh, PA) and was focused on the left eye of the mouse. A multiple infrared LED illuminator attached to the camera produced illumination and a reference corneal reflection (CR) for eye movement measurement. The eye tracker tracked the pupil center and the CR at a speed of 240 frames per second with a spatial resolution of 0.1°. Calibration was achieved by rotating the camera from the left 10° to the right 10° around the vertical axis of the eye. Following the calibration, a series of rotational and linear accelerations were delivered. To measure the steady state VOR responses, horizontal rotations were delivered at 0.2, 0.5, 1, 2, and 4 Hz (60°/s peak velocity) and horizontal translations were delivered at 0.2, 0.5, 1, and 2 Hz (0.1 g peak acceleration) along 45° right from nasal-occipital direction (i.e., the translation direction is perpendicular to the visual axis of the left eye). Step responses were not used for the LVOR because preliminary tests in WT mice showed that the transient LVOR were too small to provide reliable estimates of otolith function. To measure the transient linear VOR responses, rapid leftward or rightward 10° head rotations were delivered, which steps reached a velocity of 100°/s within 100 ms with a peak acceleration of ~1500°/s/s. At least 30 cycles/50 trials per condition were recorded. Signals related to horizontal and vertical eye position and head rotation and translation were sampled at 1 kHz at 16 bits resolution by a CED Power 1401 system (Cambridge Electronics Devices, Cambridge, UK). Eye movement responses were analyzed using Spike2 (Cambridge Electronics Devices), MatLab (MathWorks, Natick, MA) and SigmaPlot (Systat Software, San Jose, CA). Eye position signals were filtered and differentiated with a band-pass of DC to 50 Hz to obtain eye velocity signals. As described in details in Stewart et al.^35^, gains and phases of the RVORs were calculated by performing Fast Fourier Transform (FFT) on the de-saccaded eye velocity signal and head rotation velocity signal. Gains and phases of the LVORs were calculated by performing FFT on the de-saccaded eye position signal and linear head position signal. For the transient RVORs, trials in the data stream were aligned at the onset of head rotation and averaged (~50 trials per condition) to obtain low-noise estimates of eye velocity as a function of time. Gains were calculated as the ratio of eye velocity and head velocity at 80 ms after head rotation onset. VOR measurements were performed by investigators blinded to the genotype.

### Rotarod and open field assessment

Rotarod and Open Field balance assessments were performed on separate days in the Neurodevelopmental Behavioral Core at Boston Children’s Hospital. The rotarod performance test was performed over the course of two days. On day one, mice were placed in an enclosed housing on a rod that spins at a constant four r.p.m. for 5 min. If mice fell during this training session, they were immediately replaced on the rotating rod. On the second day, the trained mice were placed on the spinning rod and the spinning rate accelerated at a rate of 0.1 r.p.m. s^-1^. The length of time the animals were able to remain on the device before dropping onto the instrumented floor of the housing was displayed on a timer and recorded after each test run. A 5-min resting period was imposed between trials and a total of five trials were performed for each mouse. The open field test was conducted using a 42-cm diameter circular frame inside a sound chamber with overhead LED lighting set to 30 lux. Mice were placed one at a time inside the circular open field and allowed to explore freely and undisturbed for 5 min. Behavior was recorded and tracked using Ethovision XT, enabling measures of distance traveled, velocity, and number of rotations. A rotation was counted with the software if the mouse center-point had a cumulative turn angle of 270° over a minimum distance of 2.00 cm either counterclockwise or clockwise. Open field assessments were all conducted by investigators blinded to the genotype.

### Breeding, survival, and growth rates

For analysis of birth rate, litter survival and weight, all experimental mice had genotypes of *Tmc1*^*Δ/Δ*^*;Tmc2*^*Δ/Δ*^. Control, wild-type mice were C57BL6/J parents raising C57BL6/J pups. Male and female mice were weighed with an electronic scale at 4, 8, and 12 weeks. Females and males were housed separately and tracked over 12 weeks and were excluded if they were pregnant or had ever been pregnant. Litter frequency and survival were calculated from breeding pairs that were either *Tmc1*^*Δ/Δ*^*;Tmc2*^*Δ/Δ*^ uninjected, *Tmc1*^*Δ/Δ*^*;Tmc2*^*Δ/Δ*^ injected with sAAV-*Tmc1*, or C57BL6/J wild-type. Females were paired with *Tmc1*^*Δ/Δ*^*;Tmc2*^*Δ/Δ*^ male mice at six weeks and were housed together for 12 months. Births were manually recorded (litter size, date, and cage number). Females were retired after birthing and caring for 7 litters. To determine litter survival, litters across all breeding cages for each condition were classified as non-survival if none of the pups from the birthed litter survived to wean age (P21). Litters were classified as survival if any of the pups survived until wean age (P21). All data were recorded and evaluated using ANOVA and 2 sample independent *t*-tests for significance.

### Reporting Summary

Further information on experimental design is available in the Nature Research Reporting Summary linked to this Article.

## Supplementary Information

Supplementary Information

Description of Additional Supplementary Files

Supplementary Movie 1

Supplementary Movie 2

Supplementary Movie 3

Reporting Summary

## Data Availability

The data that support the findings of this study are available from the corresponding author upon reasonable request.
