# Publisher Correction: Improved TMC1 gene therapy restores hearing and balance in mice with genetic inner ear disorders

**DOI:** 10.1038/s41467-019-08716-x

**Published:** 2019-02-08

**Authors:** Carl A. Nist-Lund, Bifeng Pan, Amy Patterson, Yukako Asai, Tianwen Chen, Wu Zhou, Hong Zhu, Sandra Romero, Jennifer Resnik, Daniel B. Polley, Gwenaelle S. Géléoc, Jeffrey R. Holt

**Affiliations:** 1 Department of Otolaryngology and F.M. Kirby Neurobiology Center, Boston Children’s Hospital, 300 Longwood Avenue, Boston, MA 02115 USA; 2 Department of Otolaryngology, Harvard Medical School, Boston, MA 02139 USA; 3 Department of Otolaryngology and Communicative Sciences, University of Mississippi Medical Center, Jackson, MS 39216 USA; 4 Eaton-Peabody Laboratories, Massachusetts Eye and Ear Infirmary, Boston, MA 02139 USA; 5 Department of Neurology, Boston Children’s Hospital, Harvard Medical School, Boston, MA 02115 USA

Correction to: *Nature Communications*; 10.1038/s41467-018-08264-w, published online 22 Jan 2019.

The original version of this Article contained errors in Fig. 5. In panels i and j the three rightmost *x*-axis labels inadvertently read ‘Tmc1’ instead of ‘Tmc2’. These errors have been corrected in both the PDF and HTML versions of the Article.

