# Supplementary material for: Improved TMC1 gene therapy restores hearing and balance in mice with genetic inner ear disorders
Source: Nat Commun. 2019 Jan 22;10:236. doi: 10.1038/s41467-018-08264-w (PMC6342993; doi:10.1038/s41467-018-08264-w)
Supplement: Supplementary file 1 — Supplementary Information [file 41467_2018_8264_MOESM1_ESM.pdf]

Improved TMC1 gene therapy restores hearing and balance

in mice with genetic inner ear disorders

Carl A. Nist-Lund[1‡], Bifeng Pan[1,2,‡], Amy Patterson[1], Yukako Asai[1,2], Tianwen Chen[3], Wu Zhou[3],

Hong Zhu[3], Sandra Romero[4], Jennifer Resnik[2,4], Daniel B. Polley[2,4], Gwenaelle S. Géléoc[1,2]

Jeffrey R. Holt[1,2,5,]*

Corresponding author e-mail: jeffrey.holt@childrens.harvard.edu

Supplementary Information

(Contains Supplementary Figures 1 -6 with Legends)

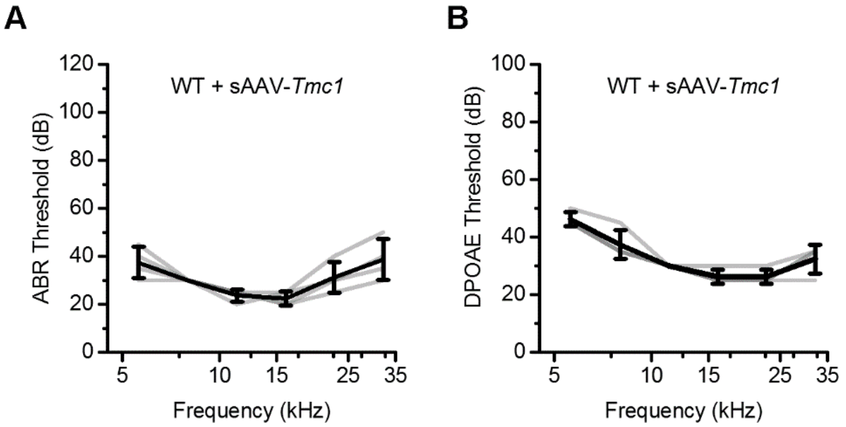

Supplementary Figure 1. sAAV-*Tmc1* injected into WT mice does not alter auditory function. **(A)** ABR thresholds plotted as a function of stimulus frequency for four WT C57BL/6 mice *Tmc1*$^{\Delta/\Delta}$ mice injected (gray traces) with 1 µL sAAV-*Tmc1* tested at P59. The black line shows mean ± S.D. ABR thresholds for the four mice, which were not statistically different from uninjected controls ($p > 0.18$). **(B)** Distortion product otoacoustic emission (DPOAE) thresholds plotted as a function of stimulus frequency for four WT C57BL/6 mice injected (gray traces) with 1 µL sAAV-*Tmc1* tested at P59. The black line shows mean ± S.D. DPOAE thresholds for the four mice, which were not elevated relative to uninjected controls.

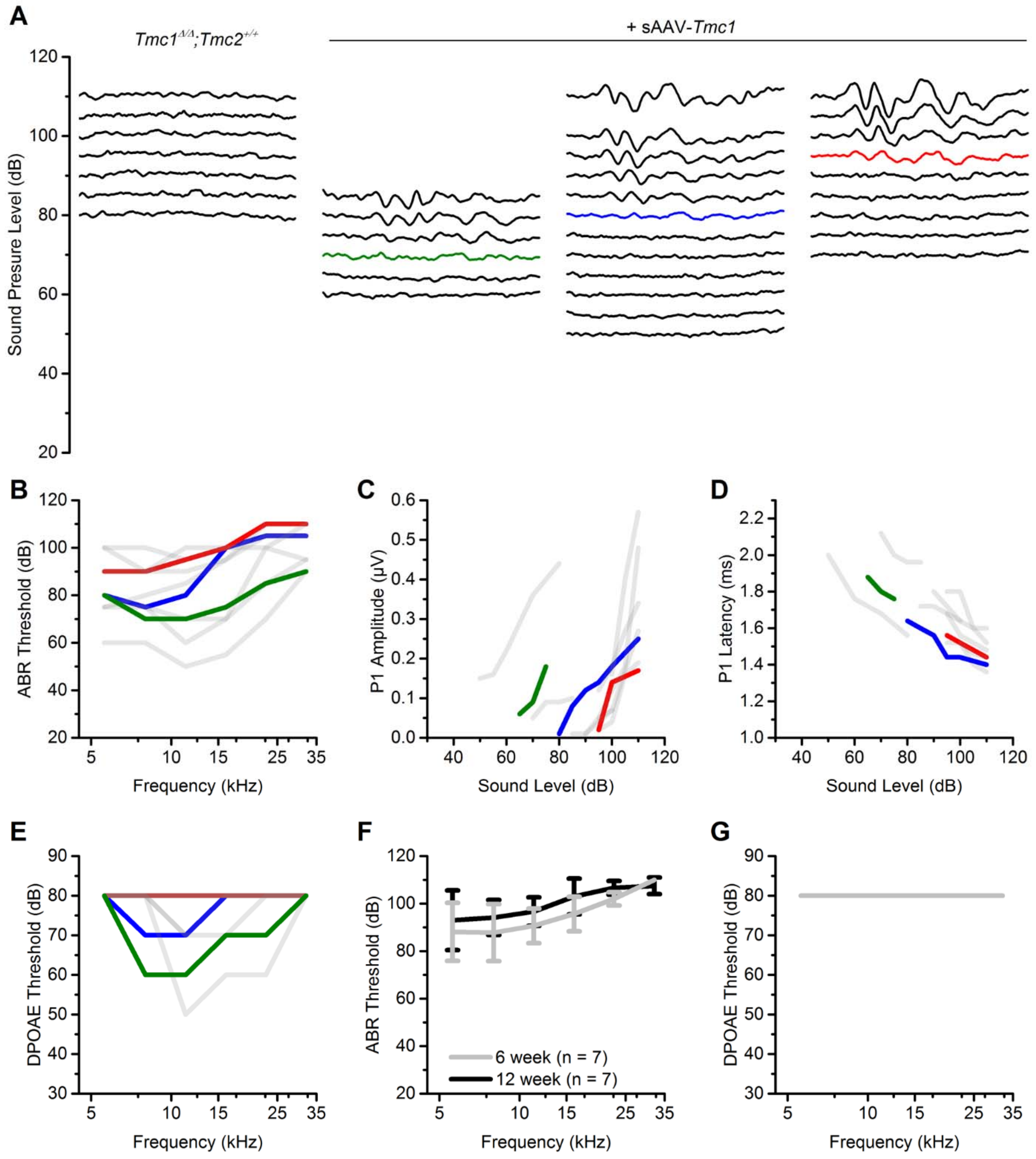

Supplementary Figure 2. sAAV-*Tmc1* restores ABRs and DPOAEs in the contralateral ears of *Tmc1*$^{\Delta/\Delta}$ mice. (A) Families of ABR waveforms recorded at P28-P30 from uninjected *Tmc1*$^{\Delta/\Delta}$ mouse (left) and from the contralateral ears of *Tmc1*$^{\Delta/\Delta}$ mice injected with sAAV-*Tmc1* shown in Figure 1 (right three traces) with variable recovery as indicated. ABRs were recorded using

11.3-kHz tone bursts at sound pressure levels increasing by 5-dB until peak amplitudes reached 0.55 µV. Thresholds determined by the presence of Peak 1 and is indicated by colored traces. Scale bar applies to all families. **(B)** ABR thresholds plotted as a function of stimulus frequency for eleven *Tmc1*$^{\Delta/\Delta}$ mice injected (gray traces) with sAAV-*Tmc1* tested at P28-P30. The contralateral of the Best (green), mean (blue) and poorest (red) recovery as determined by recovery in the injected ear are indicated. **(C)** Peak 1 amplitudes measured from 11.3-kHz ABR waveforms, as shown in (A), for eleven *Tmc1*$^{\Delta/\Delta}$ mice injected with sAAV-*Tmc1*. Colors correspond to performers indicated in (B). **(D)** Peak 1 latencies measured from 11.3-kHz ABR waveforms, as shown in (A) for eleven *Tmc1*$^{\Delta/\Delta}$ mice injected with sAAV-*Tmc1*. Colors correspond to performers indicated in (B). **(E)** DPOAE thresholds plotted as a function of stimulus frequency for the contralateral ears of eleven *Tmc1*$^{\Delta/\Delta}$ mice injected with sAAV-*Tmc1* tested at P28-P30. **(F)** Mean ± S.D. ABR thresholds plotted as a function of stimulus frequency for the contralateral ears of seven *Tmc1*$^{\Delta/\Delta}$ mice injected with sAAV-*Tmc1* tested at both 6 and 12 weeks. **(G)** DPOAE thresholds plotted as a function of stimulus frequency for the same seven *Tmc1*$^{\Delta/\Delta}$ mice injected with sAAV-*Tmc1* and tested at 6 and 12 weeks. Data points show means ± SD (*n* = number of animals).

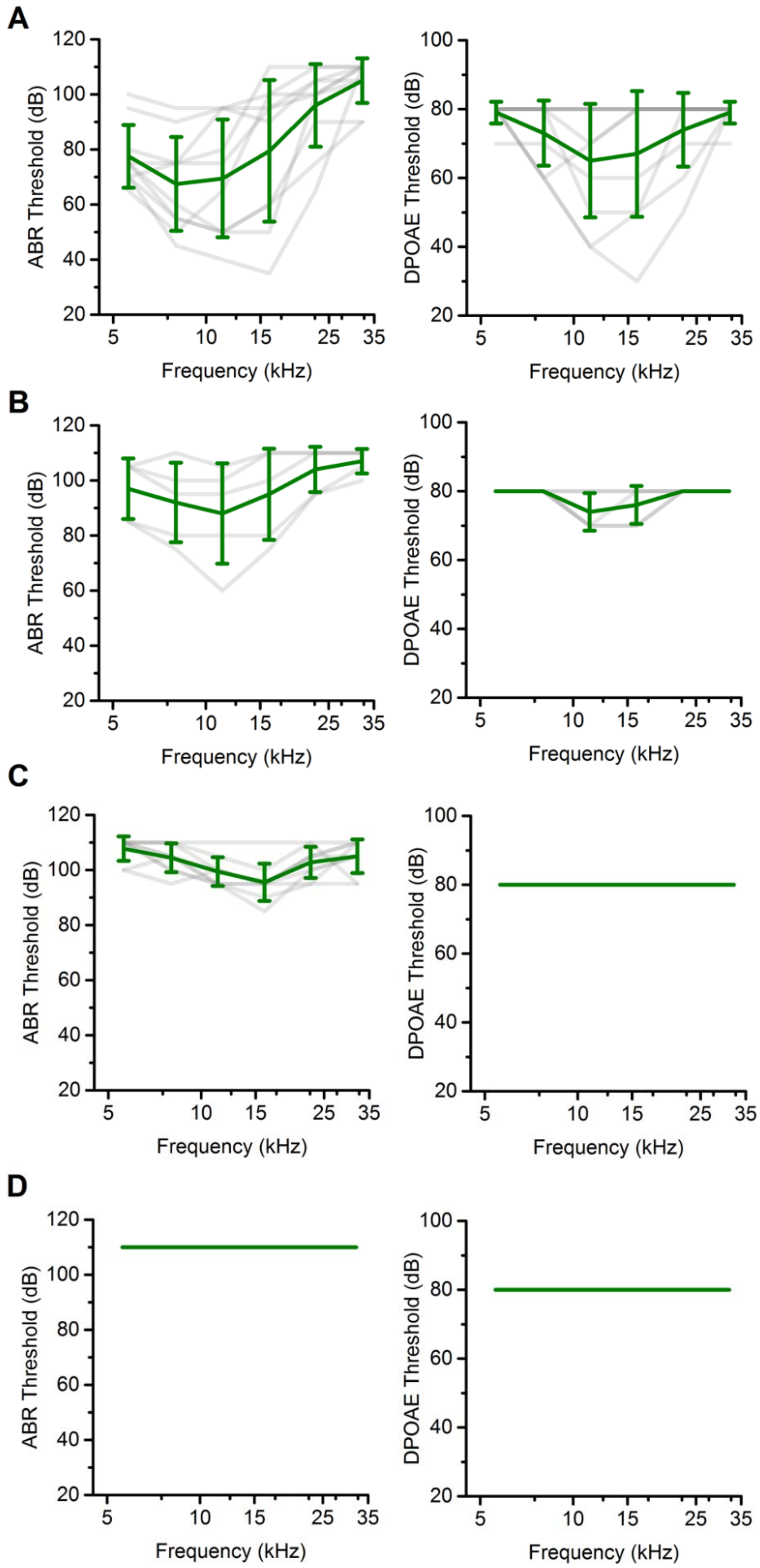
A
B
C
D
ABR Threshold (dB)
DPOAE Threshold (dB)
Frequency (kHz)
5
10
15
25
35
20
40
60
80
100
120

Supplementary Figure 3. Injection of sAAV-*Tmc1* at later time points reduces recovery of ABRs and DPOAEs. **(A)** Mean ± S.D. ABR thresholds (left) and DPOAE thresholds (right) plotted as a function of stimulus frequency for ten *Tmc1*$^{\Delta/\Delta}$ mice injected with sAAV-*Tmc1* at P1 tested at 6 weeks (P41-P43) **(B)** Mean ± S.D. ABR thresholds (left) and DPOAE thresholds (right) plotted as a function of stimulus frequency for five *Tmc1*$^{\Delta/\Delta}$ mice injected with sAAV-*Tmc1* at P4 tested at 6 weeks (P41-P42) **(C)** Mean ± S.D. ABR thresholds (left) and DPOAE thresholds (right) plotted as a function of stimulus frequency for nine *Tmc1*$^{\Delta/\Delta}$ mice injected with sAAV-*Tmc1* at P7 tested at 6 weeks (P40-P42) **(D)** Mean ± S.D. ABR thresholds (left) and DPOAE thresholds (right) plotted as a function of stimulus frequency for five *Tmc1*$^{\Delta/\Delta}$ mice injected with sAAV-*Tmc1* at P14 tested at 6 weeks (P41-P42).

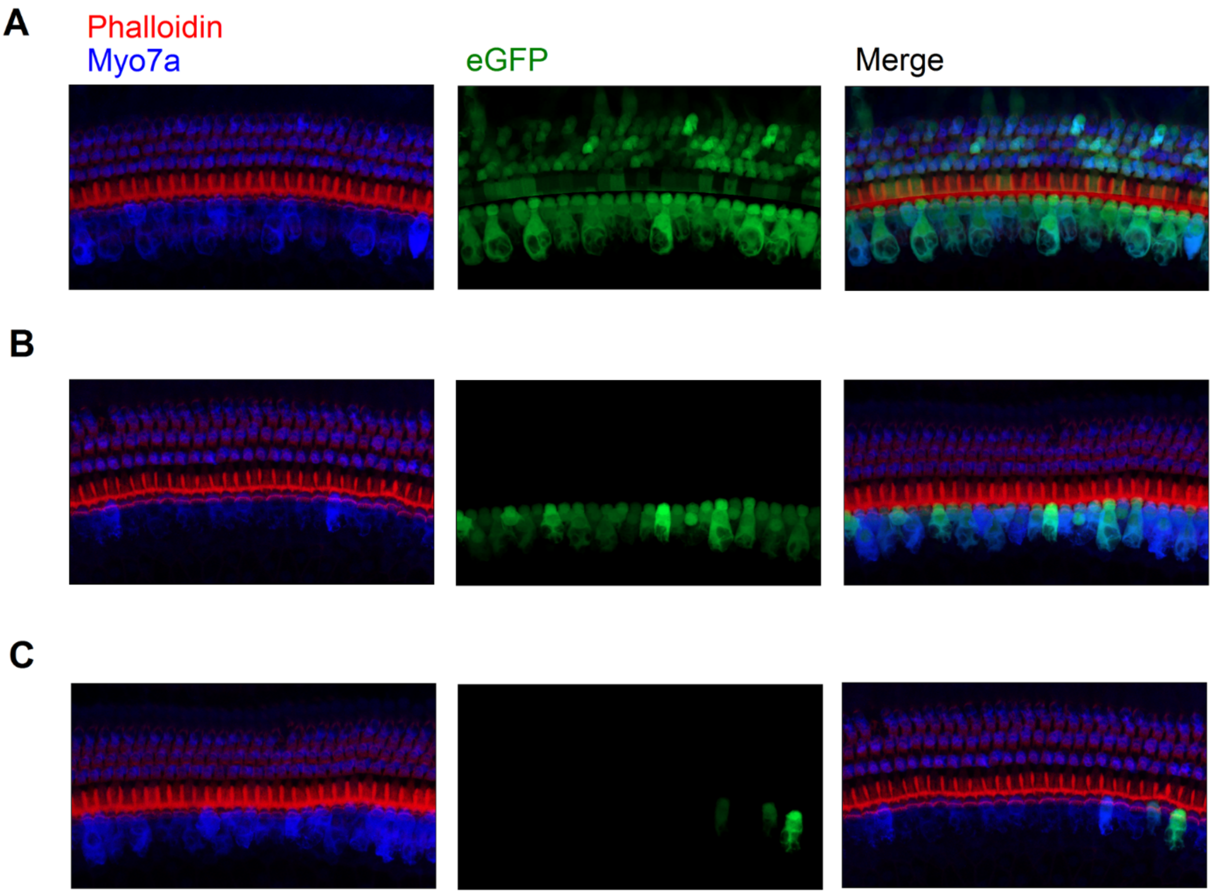

Supplementary Figure 4. Injections of sAAV-*eGFP* at P1, P7, and P14 in *Tmc1*$^{\Delta/\Delta}$ mice reduces transduction efficiency. (A) Confocal images of cochlear whole-mounts harvested at P42 from a C57BL6/J mouse injected with sAAV-*Cmv-eGFP* on P1. One hundred percent of IHCs (25/25) and 91% of OHC (74/81) were GFP-positive. The tissue was stained for Myo7a (blue) and phalloidin (red). **(B)** Confocal images of cochlear whole mounts harvest at P42 from a C57BL6/J mouse injected with sAAV-*Cmv-eGFP* on P7. The tissue was stained for Myo7a (blue) and phalloidin (red). One hundred percent of IHCs (26/26) and no OHC (0/82) were GFP-positive. **(C)** Confocal images of cochlear whole mounts harvest at P42 from a C57BL6/J mouse injected with sAAV-*Cmv-eGFP* on P14. Thirteen percent of IHCs (3/24) and no OHC (0/78) were GFP-positive. The tissue was stained for Myo7a (blue) and phalloidin (red). All images are 200 microns wide.

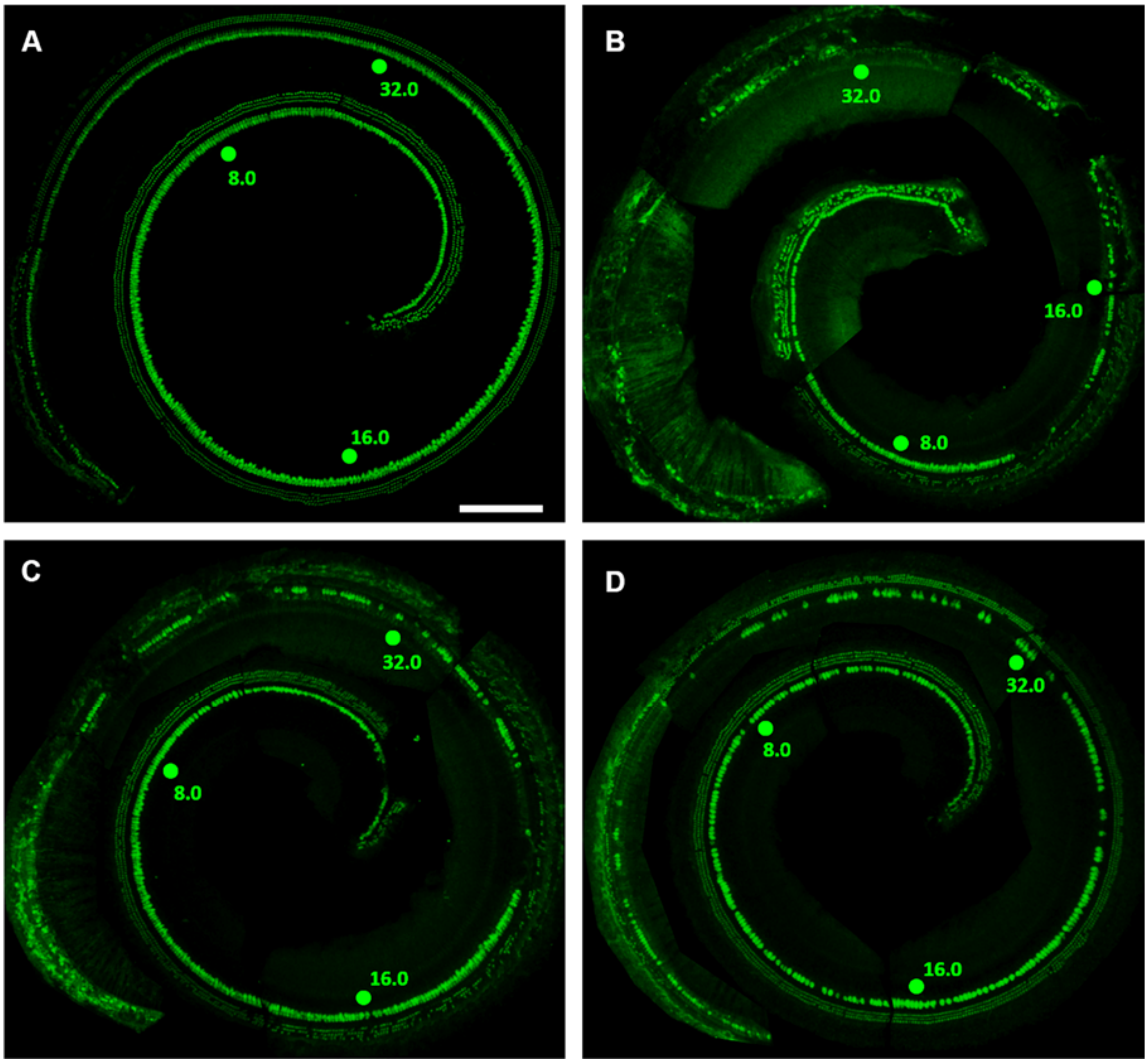

Supplementary Figure 5. Example cochlear maps and regions of interest used for hair cell survival analysis. Confocal images of cochlear whole mounts of **(A)** Uninjected C57BL6/J, **(B)** *Tmc1*$^{\Delta/\Delta}$ uninjected **(C)** *Tmc1*$^{\Delta/\Delta}$ injected sAAV-*Tmc1* with poor ABR thresholds, and **(D)** *Tmc1*$^{\Delta/\Delta}$ injected sAAV-*Tmc1* with good ABR thresholds. All mice were harvested at 12 weeks of age and dissected tissues were reconstructed to appear as a continuous tissue. Five original pieces imaged separately (extreme apex, apex, mid, base, extreme base) and reconstructed and tonotopically mapped. The tissue was stained for MYO7A (green). Scale bar = 100 microns, applies to all panels.

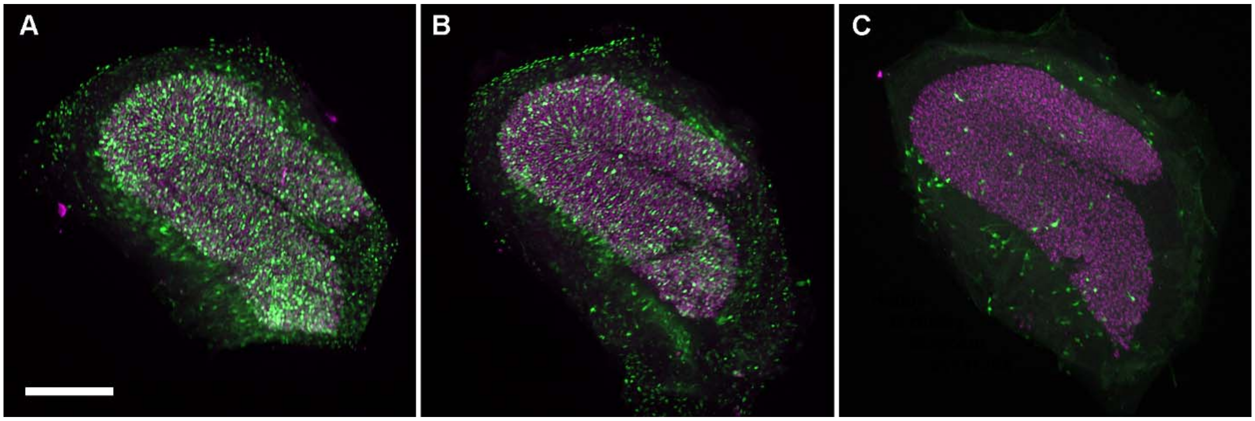

Supplementary Figure 6. Wild-type mouse saccules following inner ear injection of sAAV-*eGFP*. **(A)** Confocal image of a saccule whole-mount harvested at P28 from a C57BL6/J mouse injected with sAAV-*Cmv-eGFP* on P1. The tissue was stained for Myo7a (purple). Scale bar shows 200 microns and applies to all images. **(B)** Confocal image of a saccule whole-mount harvested at P42 from a C57BL6/J mouse injected with sAAV-*Cmv-eGFP* on P16. **(C)** Saccule whole-mount harvest at P42 from a C57BL6/J mouse injected with sAAV-*Cmv-eGFP* on P30.
