# Supplementary material for: Improved TMC1 gene therapy restores hearing and balance in mice with genetic inner ear disorders
Source: Nat Commun. 2019 Jan 22;10:236. doi: 10.1038/s41467-018-08264-w (PMC6342993; doi:10.1038/s41467-018-08264-w)
Supplement: Supplementary file 2 — Description of Additional Supplementary Files [file 41467_2018_8264_MOESM2_ESM.pdf]

Description of Additional Supplementary Files

File Name: Supplementary Movie 1
Description: Representative open field videos of a C57BL/6 control mouse.

File Name: Supplementary Movie 2
Description: Representative open field video of an uninjected Tmc1Δ/Δ;Tmc2Δ/Δ mouse.

File Name: Supplementary Movie 3
Description: Representative open field video of a Tmc1Δ/Δ;Tmc2Δ/Δ mouse injected with sAAV-Tmc1.
