# Supplementary material for: Improved TMC1 gene therapy restores hearing and balance in mice with genetic inner ear disorders
Source: Nat Commun. 2019 Jan 22;10:236. doi: 10.1038/s41467-018-08264-w (PMC6342993; doi:10.1038/s41467-018-08264-w)
Supplement: Supplementary file 6 — Reporting Summary [file 41467_2018_8264_MOESM6_ESM.pdf]

nature research

Corresponding author(s): Jeffrey R. Holt

Last updated by author(s): Dec 6, 2018

Reporting Summary

Nature Research wishes to improve the reproducibility of the work that we publish. This form provides structure for consistency and transparency in reporting. For further information on Nature Research policies, see Authors & Referees and the Editorial Policy Checklist.

Statistics

For all statistical analyses, confirm that the following items are present in the figure legend, table legend, main text, or Methods section.

n/a	Confirmed	
☐	☒	The exact sample size (n) for each experimental group/condition, given as a discrete number and unit of measurement
☐	☒	A statement on whether measurements were taken from distinct samples or whether the same sample was measured repeatedly
☐	☐	The statistical test(s) used AND whether they are one- or two-sided<br>*Only common tests should be described solely by name; describe more complex techniques in the Methods section.*
☒	☐	A description of all covariates tested
☒	☐	A description of any assumptions or corrections, such as tests of normality and adjustment for multiple comparisons
☐	☒	A full description of the statistical parameters including central tendency (e.g. means) or other basic estimates (e.g. regression coefficient) AND variation (e.g. standard deviation) or associated estimates of uncertainty (e.g. confidence intervals)
☐	☒	For null hypothesis testing, the test statistic (e.g. F, t, r) with confidence intervals, effect sizes, degrees of freedom and P value noted<br>*Give P values as exact values whenever suitable.*
☒	☐	For Bayesian analysis, information on the choice of priors and Markov chain Monte Carlo settings
☒	☐	For hierarchical and complex designs, identification of the appropriate level for tests and full reporting of outcomes
☒	☐	Estimates of effect sizes (e.g. Cohen's d, Pearson's r), indicating how they were calculated

Our web collection on statistics for biologists contains articles on many of the points above.

Software and code

Policy information about availability of computer code

Data collection	For ABR and DPOAE measurements we used Eaton-Peabody Laboratories Cochlear Function Test Suite coded in LabView. Confocal images were acquired using Zen (Zeiss). Hair cell electrophysiology data acquired using pClamp10 (Molecular Devices).
Data analysis	OriginPro 2016 (OriginLab), ZenBlue (Zeiss), Photoshop and Illustrator (Adobe).

For manuscripts utilizing custom algorithms or software that are central to the research but not yet described in published literature, software must be made available to editors/reviewers. We strongly encourage code deposition in a community repository (e.g. GitHub). See the Nature Research guidelines for submitting code & software for further information.

Data

Policy information about availability of data

All manuscripts must include a data availability statement. This statement should provide the following information, where applicable:

- Accession codes, unique identifiers, or web links for publicly available datasets
- A list of figures that have associated raw data
- A description of any restrictions on data availability

Provide your data availability statement here.

Field-specific reporting

Please select the one below that is the best fit for your research. If you are not sure, read the appropriate sections before making your selection.

☒ Life sciences ☐ Behavioural & social sciences ☐ Ecological, evolutionary & environmental sciences

Life sciences study design

All studies must disclose on these points even when the disclosure is negative.

Sample size	No sample size calculations were performed. Sample size was based on number of available mice for each condition as indicated within the manuscript.
Data exclusions	No data were excluded from the analysis.
Replication	Experiments were replicated as indicated in the manuscript.
Randomization	Mice were randomly allocated into experimental groups. Both genders were used in proportions equal to those present at birth.
Blinding	Investigators were blinded to genotype and experimental condition (injected vs non-inject).

Reporting for specific materials, systems and methods

We require information from authors about some types of materials, experimental systems and methods used in many studies. Here, indicate whether each material, system or method listed is relevant to your study. If you are not sure if a list item applies to your research, read the appropriate section before selecting a response.

Materials & experimental systems

n/a	Involved in the study	
☐	☒	Antibodies
☒	☐	Eukaryotic cell lines
☒	☐	Palaeontology
☐	☒	Animals and other organisms
☒	☐	Human research participants
☒	☐	Clinical data

Methods

n/a	Involved in the study	
☒	☐	ChIP-seq
☒	☐	Flow cytometry
☒	☐	MRI-based neuroimaging

Antibodies

Antibodies used	Primary antibody: a rabbit anti-Myosin VIIa primary antibody (Proteus Biosciences, PRODUCT #: 25-6790), Secondary antibody: donkey anti-rabbit antibody conjugated to AlexaFluor555 (Life Technologies). Both were validated by the vendors.
Validation	Primary Antibody validated as described by:<br>Hasson, T. et al. (1995), Proc. Natl. Acad. Sci. 92:9815-9819.<br>Hasson, T. et al. (1997), Journal of Cell Biology 137:1287-1307.<br>Hasson, T. et al. (1997), Cell Motil. Cytoskel. 37:127-138.<br>Hasson, T. et al. (2005), Cell Motil. Cytoskel. 62:13-26.

Animals and other organisms

Policy information about studies involving animals; ARRIVE guidelines recommended for reporting animal research

Laboratory animals	Tmc1-null and Tmc2-null mice on C57B/6-129 background obtained from Jackson Labs (019146 - B6.129-Tmc1tm1.1Ajg/J; 019147 - B6.129-Tmc2tm1.1Ajg/J)
Wild animals	C57B/6 mice obtained from Jackson Labs
Field-collected samples	The study did not involve samples collected from the field.
Ethics oversight	Protocols were approved by the Institutional Animal Care and Use Committee (protocols #18-01-3610R and #17-03-3396R) at Boston Children's Hospital.

Note that full information on the approval of the study protocol must also be provided in the manuscript.
